# The association between red blood cell distribution width-to-albumin ratio and *Helicobacter pylori* seropositivity in U.S. adults: an observational study from NHANES 1999–2000 with external validation

**DOI:** 10.3389/fnut.2026.1781850

**Published:** 2026-05-08

**Authors:** Jinfang Zeng, Shu Yang, Jinjin Jian, Minmin Zhu, Jingbin Ni, Meng Zhang

**Affiliations:** 1 Department of Anesthesiology and Pain Medicine, Wuxi No. 2 People’s Hospital (Jiangnan University Medical Center), Wuxi, China; 2 Wuxi School of Medicine, Jiangnan University, Wuxi, China; 3 Department of Anesthesiology, Affiliated Hospital of Jiangnan University, Wuxi, China; 4 Department of Gastroenterology, Wuxi No. 2 People’s Hospital (Jiangnan University Medical Center), Wuxi, China

**Keywords:** cross-sectional study, *Helicobacter pylori*, inflammation, NHANES, red blood cell distribution width-to-albumin ratio, seropositivity

## Abstract

**Objectives:**

*Helicobacter pylori* (Hp) seropositivity, linked to inflammatory morbidity, may reflect host inflammation-nutrition imbalance. The red blood cell distribution width-to-albumin ratio (RAR) has been proposed as a composite marker reflecting this axis. Using NHANES data and an external validation cohort of 250 hospital patients, we aimed to examine the association between RAR and *H. pylori* seropositivity.

**Methods:**

This study included U.S. adults from NHANES 1999–2000 and an external validation cohort of 250 hospital patients. Survey-weighted logistic regression assessed the association between RAR and *H. pylori* seropositivity. Restricted cubic splines (RCS) evaluated non-linearity. In addition, we performed a series of analyses, including restricting the range of RAR to limit inter-queue overlap, excluding subjects with clinical anemia, conducting sensitivity analyses by adjusting spline function parameters and handling extreme values, as well as subgroup analyses.

**Results:**

In the main adult dataset of NHANES 1999–2000, RCS regression analysis revealed a significant nonlinear association between RAR and Hp seropositivity rate (overall test *p* < 0.001; nonlinear test *p* < 0.001), with the inflection point at RAR = 2.85. Below this point, higher RAR was associated with greater odds of seropositivity (OR = 3.59; 95% CI: 1.76–7.31); above it, higher RAR related to lower odds (OR = 0.73; 95% CI: 0.55–0.97). RCS analysis of the external validation cohort showed a linear RAR-Hp seropositivity association (overall *p* = 0.457; nonlinear *p* = 0.937). Most RAR values clustered above the inflection point (2.85), potentially reducing statistical power. Restricting both cohorts to RAR ≥ 2.85 yielded consistent linear downward trends, with no significant nonlinearity in either cohort, indicating the validation cohort reflects post-inflection local relationships. In categorical analyses, the third RAR quartile remained positively associated after full adjustment, whereas the highest quartile did not, consistent with nonlinearity. Subgroup analyses showed no significant associations overall, though potential heterogeneity was observed by hypertension (*p* for interaction = 0.040) and age (*p* for interaction = 0.046).

**Conclusion:**

A statistically significant non-linear association pattern was observed between RAR and the seropositivity rate of *Helicobacter pylori* in NHANES; however, this association was sensitive to exposure distribution and could be partially explained by hematological factors. The findings were not replicated in the external cohort, and the overall evidence does not support a robust or clinically applicable association. RAR may reflect the inflammation-nutrition microenvironment associated with *Helicobacter pylori* exposure, rather than functioning as an independent predictive biomarker. Prospective studies are warranted to elucidate the causal relationship and clinical implications.

## 1 Introduction

*Helicobacter pylori* is a helical, Gram-negative microorganism that persistently inhabits the gastric mucosa (1). Its prevalence is widespread, affecting nearly 50% of the global population, and it has been designated a Group I carcinogen by the WHO (2). The bacterium is a key contributor to chronic gastritis, peptic ulcers, MALT lymphoma, and gastric cancer excluding the cardia region (1), and has also been associated with various systemic disorders, including those affecting the metabolic, cardiovascular, skeletal, and nervous systems (3). Population burden includes dyspepsia, ulcer bleeding, iron-deficiency anemia, cancer mortality, and substantial costs (4).

Eradication with triple or bismuth-based quadruple therapy is effective (1), yet antimicrobial resistance (5), imperfect adherence, and recurrence limit impact; no vaccine exists (6). Strengthened prevention, resistance-guided treatment, and simple risk-stratifying biomarkers are urgently needed (2, 7).

Emerging data indicate that systemic inflammation underlies *Helicobacter pylori* seropositivity, with infected adults exhibiting higher circulating inflammatory markers such as high-sensitivity C-reactive protein, consistent with extra-gastric immune activation (8). In hematologic profiling, *H. pylori* infection has been linked to shifts in erythrocyte indices, including increased red cell distribution width (RDW), supporting an inflammation-related dyserythropoiesis signal in cross-sectional comparisons (9). Concurrently, HP-positive individuals tend to have lower serum albumin and reduced albumin/globulin ratios, implicating a nutrition–inflammation axis that conventional single markers may not fully capture (10). However, RDW and albumin alone are vulnerable to confounding by acute illness, comorbidities, and assay variability, limiting interpretability in population surveillance. To address these constraints, RAR integrates two routine, low-cost measures into a composite index that more holistically reflects inflammatory and nutritional status;

Over the past years, studies have increasingly used RAR as a low-cost proxy of the inflammation–nutrition axis with relevance to digestive disease. In acute pancreatitis, RAR consistently tracks illness severity and adverse outcomes, complementing established scores in retrospective and meta-analytic work (11). Beyond the pancreas, cross-sectional analyses link higher RAR with greater colorectal cancer risk in population datasets, suggesting utility for epidemiologic stratification rather than diagnosis (12). In liver-related contexts, elevated RAR associates with mortality among critically ill patients with NAFLD, aligning with observations that deranged erythropoiesis and hypoalbuminemia portend poor outcomes (13). Mechanistically, RDW captures inflammation-related dyserythropoiesis and oxidative stress, while albumin—being a negative acute-phase reactant—reflects protein–energy status and capillary leak; their ratio integrates these complementary signals into a more stable composite than cytokines alone (14). Evidence from general cohorts further shows graded RAR–mortality associations, reinforcing its prognostic breadth and scalability for surveys (15).

Addressing gaps in infection-focused epidemiology, we conducted a cross-sectional analysis of nationally representative NHANES data to assess whether RAR is associated with *Helicobacter pylori* seropositivity—the first NHANES-based evaluation of this relationship in U.S. adults.

## 2 Materials and methods

### 2.1 Study population

We conducted a cross-sectional analysis using data from NHANES, a program managed by the CDC’s National Center for Health Statistics. NHANES utilizes a complex, multistage probability sampling method to represent the non-institutionalized civilian population of the United States. Data collection includes standardized in-home interviews, along with physical exams and lab tests conducted in mobile examination units. For the present study, we pooled the 1999–2000 cycles and included participants with complete measurements of red blood cell distribution width, serum albumin, and *Helicobacter pylori* serology, as well as relevant demographic and lifestyle covariates. The NHANES protocol received approval from the Research Ethics Review Board of the National Center for Health Statistics. All participants gave written informed consent prior to participation. Comprehensive details about the survey methodology can be accessed on the official NHANES website (16).^1^

**Figure 1:**
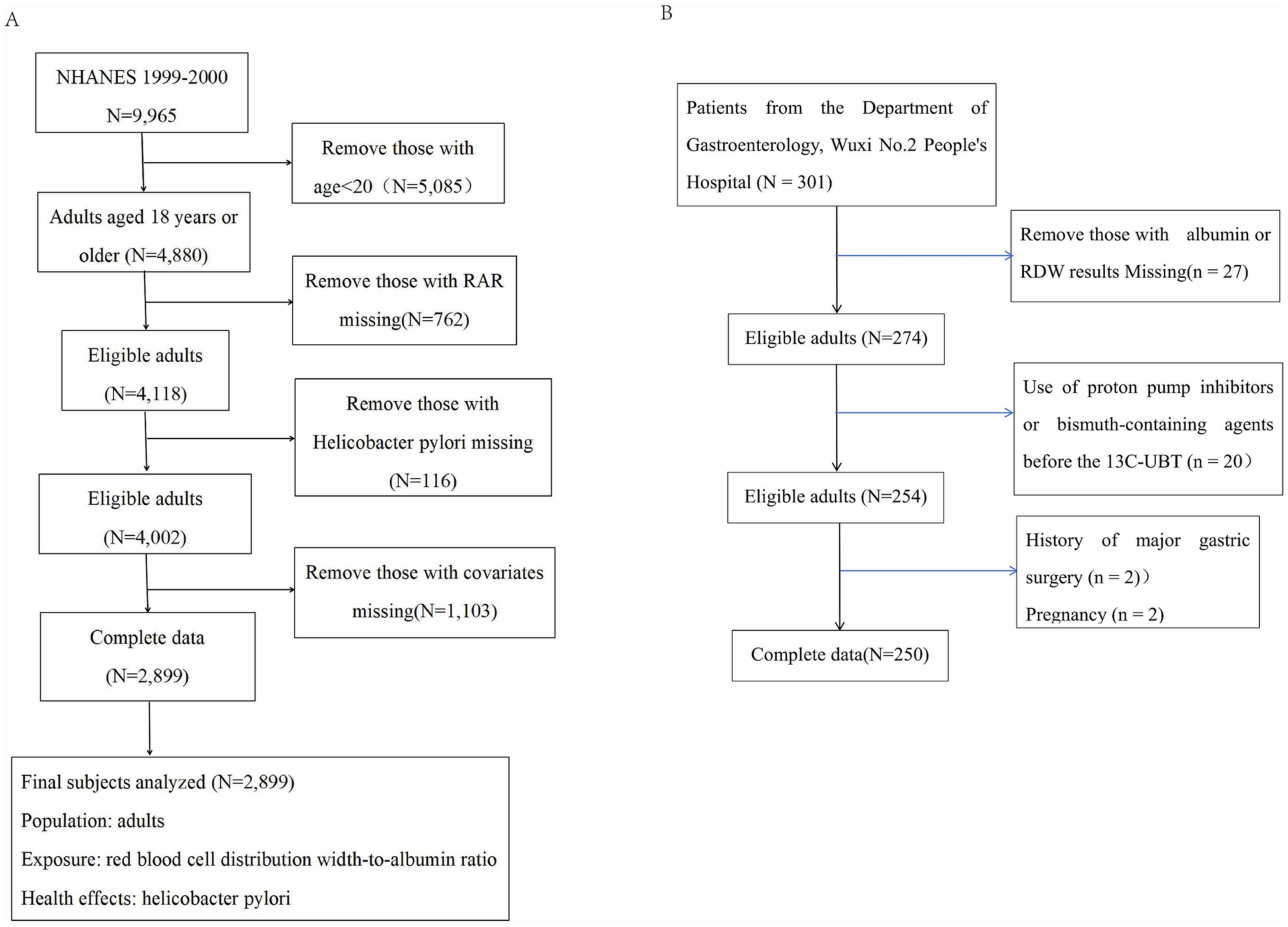
Flow diagram of the inclusion criteria **(A)** and exclusion criteria **(B)**.

For this analysis, we used NHANES 1999–2000 data (initial *N* = 9,965). We excluded participants younger than 20 years (*n* = 5,085), those missing RAR values (*n* = 762), individuals without *Helicobacter pylori* serology (*n* = 116), and those lacking key covariates (*n* = 1,103). The final analytic cohort comprised 2,899 adults for evaluating the association between RAR and *H. pylori* seropositivity (Figure 1A).

#### 2.1.1 External validation cohort

For external validation, an independent hospital-based cohort was assembled at Wuxi No. 2 People’s Hospital by retrospectively reviewing the electronic medical record system. Adult patients who received a ^13C urea breath test (^13C-UBT) for *Helicobacter pylori* detection from January 1, 2025 to December 31, 2025 were consecutively identified. Data on demographics, clinical characteristics, and laboratory findings were extracted from the medical records. Only patients with complete information on RDW, serum albumin, and ^13C-UBT results were considered eligible. The final validation cohort was defined after applying the prespecified inclusion and exclusion criteria.

Inclusion and exclusion criteria.

Patients were eligible for inclusion in the external validation cohort if they were aged 18 years or older, underwent a ^13C urea breath test (^13C-UBT) for *Helicobacter pylori* detection between January 1, 2025 and December 31, 2025, and had available measurements ofRDW and serum albumin obtained during the same visit or within a predefined interval relative to the ^13C-UBT. In addition, complete baseline demographic and laboratory data required for the analysis were necessary for inclusion.

Patients were excluded if they were younger than 18 years, lacked ^13C-UBT results, RDW values, or serum albumin data, or had a documented history of prior *H. pylori* eradication therapy. Those with recent exposure to antibiotics, proton pump inhibitors, or bismuth-containing agents before the ^13C-UBT were also excluded because these medications could affect test accuracy. Additional exclusion criteria included major hematologic disorders, active infection, malignancy, severe hepatic or renal dysfunction, other conditions likely to markedly alter RDW or serum albumin levels, a history of major gastric surgery, and pregnancy. For patients with more than one eligible record during the study period, only the first complete record was retained for analysis. The detailed flowchart of inclusion and exclusion is presented in Figure 1B.

### 2.2 *Helicobacter pylori* seropositivity

In NHANES, serum samples were collected via venipuncture, then aliquoted and preserved at −80 °C until batch analysis at the University of Washington. Levels of anti–*Helicobacter pylori* immunoglobulin G (IgG) were measured using a commercial ELISA kit (Wampole Laboratories, Cranbury, NJ), following established standardized procedures (17). Serostatus was defined *a priori* using established optical-density (OD) thresholds: seropositive (OD ≥ 1.1) and seronegative (OD < 0.9); specimens with equivocal values (0.9–1.1) were excluded to minimize misclassification and preserve analytic validity (18).

### 2.3 Measurements of RAR

RAR was defined as RDW (%) divided by serum albumin (g/dL) (19). RDW was obtained from peripheral venous blood as part of the complete blood count and measured on a Coulter DxH 800 hematology analyzer in accordance with standardized NHANES procedures at the Mobile Examination Centers. Serum albumin was quantified using the bromocresol purple (BCP) dye-binding assay, with the absorbance of the albumin–BCP complex recorded at 600 nm by spectrophotometry. For statistical analyses, RAR was modeled both as a continuous variable and as quartiles derived from the distribution of the analytic sample, with the lowest quartile (Q1) serving as the reference. Detailed assay protocols are provided in the NHANES Laboratory/Medical Technicians Procedure Manual (20).

### 2.4 Covariates

Covariates were selected *a priori* based on prior literature and clinical plausibility, with emphasis on variables that could be associated with both RAR and *H. pylori* seropositivity and therefore act as potential confounders. Guided by previous studies and the NHANES methodology, potential confounders were identified in advance across several domains, including demographic, socioeconomic, behavioral, anthropometric, and clinical factors. Socioeconomic status was assessed first, based on participants’ highest level of education—categorized as less than high school, high school or GED, and more than high school—and their marital status, which was classified as married or cohabiting, widowed, divorced or separated, and never married. Demographic information included age, recorded as a continuous variable; sex, defined as either male or female; and race or ethnicity, which was grouped into five categories: Non-Hispanic White, Non-Hispanic Black, Mexican American, Other Hispanic, and Other or Multiracial (21), and PIR categorized as ≤130%, 130–350%, or >350% of the federal poverty level. Behavioral factors comprised smoking status—classified as smoker if ≥100 lifetime cigarettes and nonsmoker otherwise—and alcohol intake, defined as drinker if ≥12 alcoholic beverages were reported in the prior year and nondrinker if <12 or never. BMI was calculated using measured height and weight, then categorized based on World Health Organization guidelines. Participants were classified as having normal weight if their BMI was below 25 kg/m^2^, overweight if it ranged from 25 to 30 kg/m^2^, and obese if it was 30 kg/m^2^ or higher. Clinical comorbidities were determined using standardized NHANES protocols and included hypertension, diabetes, coronary heart disease, and stroke. These conditions were identified through self-reported physician diagnoses and/or the reported use of medications specific to each disease (22). All covariates were defined and measured following NHANES guidelines to ensure comparability and data quality. Prior to model fitting, we evaluated multicollinearity among covariates—sex, age, race/ethnicity, education level, marital status, PIR, alcohol use, BMI, hypertension, diabetes, coronary heart disease, stroke, and smoking—using variance inflation factors (VIFs). Categorical variables were entered as dummy indicators with the reference level omitted, and continuous variables were modeled as linear terms. After listwise deletion of observations with missing predictor data, all VIFs were below conventional thresholds for concern (maximum VIF = 1.93; VIF > 10 prespecified as indicative of serious collinearity), indicating that multicollinearity was not a material issue in our analyses (Supplementary Table 1).

### 2.5 Statistical analyses

All statistical analyses were conducted using R software version 4.3.0, EmpowerStats version 5.0, and Zstats version 1.0, which is accessible at www.zstats.net. To generate nationally representative estimates, the analysis incorporated sample weights, strata, and primary sampling units in accordance with the NHANES complex, multistage probability sampling framework. These adjustments accounted for unequal probabilities of selection, nonresponse bias, and post-stratification. Continuous variables were summarized using weighted means along with their standard errors, while categorical variables were described as weighted proportions. To assess the relationship between RAR and *Helicobacter pylori* seropositivity, survey-weighted logistic regression models were used to estimate odds ratios and corresponding 95% confidence intervals. RAR was evaluated both as a continuous variable, assessed per one standard deviation increase, and as a categorical variable divided into quartiles, using the lowest quartile as the reference group. Three models were constructed to evaluate the association. Three models were constructed to evaluate the association between RAR and *H. pylori* seropositivity using a sequential adjustment strategy. The unadjusted model estimated the crude association. Model I adjusted for age, sex, and race/ethnicity, because these basic demographic variables are strongly associated with both inflammatory and nutritional status and *H. pylori* seropositivity. Model II further included education level, marital status, poverty-income ratio, alcohol consumption, smoking status, BMI, hypertension, diabetes, coronary heart disease, and stroke, as these variables were considered *a priori* potential confounders on epidemiologic and clinical grounds. This stepwise approach was used to show the extent to which the observed association was explained by successive adjustment for demographic, socioeconomic, behavioral, anthropometric, and clinical factors, rather than to imply a causal sequence among covariates. To assess potential nonlinear relationships between RAR and *H. pylori* seropositivity, restricted cubic spline regression was employed with knots placed at the 10th, 50th, and 90th percentiles of RAR distribution. Two-piecewise logistic regression was then used to identify possible inflection points, selected based on maximum model likelihood. Likelihood ratio tests were conducted to compare the fit between linear and piecewise models. Subgroup analyses assessed effect modification by sex, age (<60 vs. ≥60 years), race/ethnicity, BMI, smoking, alcohol use, diabetes, hypertension, coronary heart disease, stroke, poverty-income ratio, serum iron, and C-reactive protein, with interactions evaluated via likelihood ratio tests.

We conducted an exploratory analysis of the discriminative performance of RAR for *H. pylori* seropositivity using ROC curves with 2,000-bootstrap 95% CIs and the Youden index to identify optimal cut-offs, accounting for the NHANES complex survey design where applicable. Given the etiologic focus of this study, these diagnostic comparisons are presented in the Supplementary material.

We assessed robustness using a series of sensitivity analyses based on multivariable logistic regression with RAR as the exposure. In the primary model, RAR was standardized (per SD). We repeated the analysis after excluding participants with CRP > 10 mg/dL; after excluding those with ALT, AST, or GGT ≥ 95th percentile; and after trimming RAR to the 1st–99th percentiles. We also modeled ln(RAR). To evaluate dose–response, RAR was grouped into quartiles (Q1 as reference) and a trend across quartiles was tested. All models used the same covariate adjustment as the main analysis, and two-sided *α* = 0.05 was applied. Results are shown in Supplementary Table 3.

We applied two-sided tests and considered *p* values under 0.05 statistically significant. Multiple-comparison adjustments were omitted since the subgroup analyses were exploratory, aimed at hypothesis generation.

Participants with missing exposure, outcome, or covariate data were excluded from the primary analyses, and all regression models were therefore based on complete-case data.

## 3 Results

### 3.1 Characteristics of the participants

In this cross-sectional NHANES sample, 2,899 adults were included in the analysis, comprising 1,648 participants who were *H. pylori* seronegative and 1,251 who were seropositive. Table 1 summarizes baseline characteristics by serostatus. The overall mean age was 49.46 ± 18.63 years; participants with *H. pylori* seropositivity were older than those who were seronegative (52.88 ± 17.90 vs. 46.86 ± 18.77 years, *p* < 0.001). The distribution of sex did not differ statistically between groups (*p* = 0.162). Marked differences were observed across race/ethnicity (*p* < 0.001): Mexican American (40.13% vs. 15.59%) and other Hispanic (9.03% vs. 4.31%) participants were more prevalent in the seropositive group, whereas non-Hispanic White participants were less common (25.74% vs. 63.71%); the proportion of non-Hispanic Black participants was higher among those seropositive (21.74% vs. 13.05%). Educational attainment also differed (*p* < 0.001), with a higher proportion of individuals with less than high school education in the seropositive group (55.16% vs. 21.97%) and a lower proportion with above–high school education (25.74% vs. 53.03%). Marital status distributions varied (*p* < 0.001): married/living with partner (64.59% vs. 61.89%), widowed/divorced/separated (23.90% vs. 20.15%), and never married (11.51% vs. 17.96%). Socioeconomic status, as indexed by PIR, showed clear gradients (*p* < 0.001): PIR < 1.3 was more common in the seropositive group (40.21% vs. 22.03%), whereas PIR ≥ 3.5 was less common (19.82% vs. 41.57%). BMI categories differed modestly (*p* = 0.021), with higher proportions of overweight (25–30 kg/m^2^, 36.85% vs. 34.71%) and obesity (≥ 30 kg/m^2^; 34.29% vs. 31.61%) in the seropositive group and a lower proportion with BMI < 25 kg/m^2^ (28.86% vs. 33.68%).

**Table 1:** Baseline characteristics of participants with *H. pylori* seronegative or H. pylori seropositive.

Characteristics	Overall (*n* = 2,899)	*H. pylori* seronegative (*n* = 1,648)	*H. pylori* seropositive (*n* = 1,251)	*p*-value
Age	49.46 ± 18.63	46.86 ± 18.77	52.88 ± 17.90	<0.001
Gender				0.162
Male	1,389 (47.91%)	771 (46.78%)	618 (49.40%)	
Female	1,510 (52.09%)	877 (53.22%)	633 (50.60%)	
Race				<0.001
Mexican American	759 (26.18%)	257 (15.59%)	502 (40.13%)	
Other Hispanic	184 (6.35%)	71 (4.31%)	113 (9.03%)	
Non-Hispanic White	1,372 (47.33%)	1,050 (63.71%)	322 (25.74%)	
Non-Hispanic Black	487 (16.80%)	215 (13.05%)	272 (21.74%)	
Other race	97 (3.35%)	55 (3.34%)	42 (3.36%)	
Education level				<0.001
Less than high school	1,052 (36.29%)	362 (21.97%)	690 (55.16%)	
High school	651 (22.46%)	412 (25.00%)	239 (19.10%)	
Above high school	1,196 (41.26%)	874 (53.03%)	322 (25.74%)	
Marital status				<0.001
Married/Living with partner	1828 (63.06%)	1,020 (61.89%)	808 (64.59%)	
Widowed/Divorced/Separated	631 (21.77%)	332 (20.15%)	299 (23.90%)	
Never married	440 (15.18%)	296 (17.96%)	144 (11.51%)	
PIR				<0.001
<1.3	866 (29.87%)	363 (22.03%)	503 (40.21%)	
1.3–3.5	1,100 (37.94%)	600 (36.41%)	500 (39.97%)	
≥3.5	933 (32.18%)	685 (41.57%)	248 (19.82%)	
BMI				0.021
<25	916 (31.60%)	555 (33.68%)	361 (28.86%)	
25–30	1,033 (35.63%)	572 (34.71%)	461 (36.85%)	
≥30	950 (32.77%)	521 (31.61%)	429 (34.29%)	
C-reactive protein(mg/dL)	0.50 ± 0.94	0.49 ± 1.04	0.52 ± 0.78	<0.001
ALT (U/L)	27.07 ± 34.53	26.59 ± 35.83	27.69 ± 32.74	0.099
AST (U/L)	25.51 ± 25.33	25.21 ± 28.44	25.89 ± 20.53	0.004
Cholesterol, total (mmol/L)	5.13 ± 1.03	5.12 ± 1.02	5.16 ± 1.04	0.167
GGT(U/L)	32.40 ± 40.67	30.02 ± 34.62	35.53 ± 47.30	<0.001
Glucose (mmol/L)	5.42 ± 1.99	5.24 ± 1.70	5.65 ± 2.29	<0.001
Iron (umol/L)	15.77 ± 6.34	16.04 ± 6.48	15.41 ± 6.14	0.022
Triglycerides (mmol/L)	1.64 ± 1.15	1.58 ± 1.09	1.73 ± 1.22	<0.001
Creatinine (mg/dL)	0.76 ± 0.60	0.76 ± 0.60	0.75 ± 0.59	0.632
Alcohol				0.017
Yes	1967 (67.85%)	1,148 (69.66%)	819 (65.47%)	
No	932 (32.15%)	500 (30.34%)	432 (34.53%)	
Smoking				0.014
Yes	1,386 (47.81%)	755 (45.81%)	631 (50.44%)	
No	1,513 (52.19%)	893 (54.19%)	620 (49.56%)	
Hypertension				<0.001
Yes	870 (30.01%)	454 (27.55%)	416 (33.25%)	
No	2029 (69.99%)	1,194 (72.45%)	835 (66.75%)	
Diabetes				<0.001
Yes	296 (10.21%)	120 (7.28%)	176 (14.07%)	
No	2,603 (89.79%)	1,528 (92.72%)	1,075 (85.93%)	
Coronary heart disease				0.545
Yes	111 (3.83%)	60 (3.64%)	51 (4.08%)	
No	2,788 (96.17%)	1,588 (96.36%)	1,200 (95.92%)	
Stroke				0.006
Yes	87 (3.00%)	37 (2.25%)	50 (4.00%)	
No	2,812 (97.00%)	1,611 (97.75%)	1,201 (96.00%)	
RAR	2.90 ± 0.40	2.87 ± 0.41	2.94 ± 0.38	<0.001
RAR group				<0.001
Q1	724 (24.97%)	490 (29.73%)	234 (18.71%)	
Q2	722 (24.91%)	412 (25.00%)	310 (24.78%)	
Q3	726 (25.04%)	359 (21.78%)	367 (29.34%)	
Q4	727 (25.08%)	387 (23.48%)	340 (27.18%)	

PIR, Poverty Income Ratio; BMI, Body Mass Index; RAR, Red Blood Cell Distribution Width-to-Albumin Ratio.

Biochemical and clinical profiles were also distinct. Compared with seronegative participants, those seropositive had higher levels of C-reactive protein (0.52 ± 0.78 vs. 0.49 ± 1.04 mg/dL; *p* < 0.001), aspartate aminotransferase (25.89 ± 20.53 vs. 25.21 ± 28.44 U/L; *p* = 0.004), *γ*-glutamyltransferase (35.53 ± 47.30 vs. 30.02 ± 34.62 U/L; *p* < 0.001), fasting glucose (5.65 ± 2.29 vs. 5.24 ± 1.70 mmol/L; *p* < 0.001), and triglycerides (1.73 ± 1.22 vs. 1.58 ± 1.09 mmol/L; *p* < 0.001), but lower serum iron (15.41 ± 6.14 vs. 16.04 ± 6.48 μmol/L; *p* = 0.022). Differences were not observed for alanine aminotransferase (*p* = 0.099), total cholesterol (*p* = 0.167), or creatinine (*p* = 0.632). Regarding lifestyle factors, current alcohol use was less common among seropositive participants (65.47% vs. 69.66%; *p* = 0.017), whereas current smoking was more common (50.44% vs. 45.81%; *p* = 0.014). In terms of comorbid conditions, the prevalence of hypertension (33.25% vs. 27.55%; *p* < 0.001), diabetes (14.07% vs. 7.28%; *p* < 0.001), and stroke (4.00% vs. 2.25%; *p* = 0.006) was higher among the seropositive group; no difference was detected for coronary heart disease (*p* = 0.545).

With respect to the exposure of interest, RAR was higher in the seropositive group (2.94 ± 0.38) than in the seronegative group (2.87 ± 0.41; *p* < 0.001), and the distribution across RAR quartiles differed (*p* < 0.001), with seropositive participants more frequently represented in the higher quartiles (Q3–Q4) and less frequently in Q1 (Q1: 18.71% vs. 29.73%; Q3: 29.34% vs. 21.78%; Q4: 27.18% vs. 23.48%). These descriptive, cross-sectional comparisons indicate that *H. pylori* seropositivity co-occurs with older age, lower socioeconomic and educational indicators, adverse cardiometabolic profiles, and higher RAR, without implying directionality or causation.

**Figure 2:**
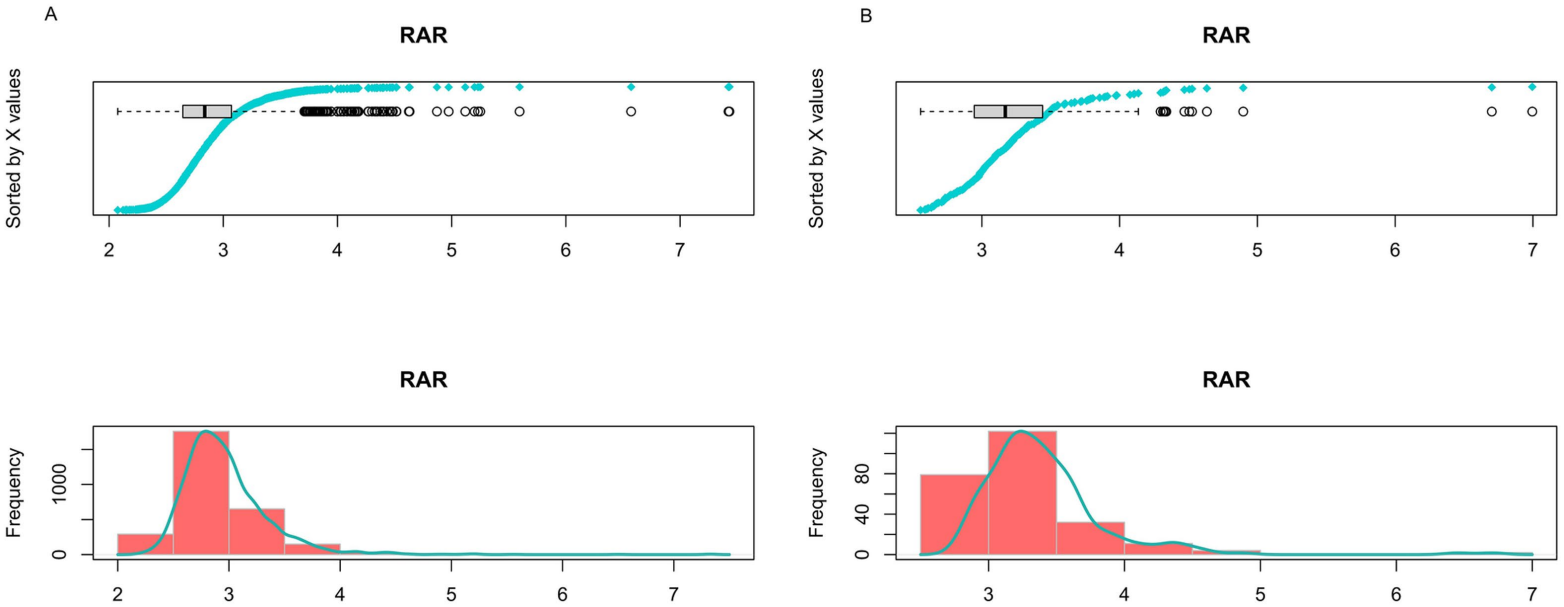
Distribution of RAR in the NHANES discovery cohort **(A)** and external validation cohort **(B)**.

Supplementary Table 2 presents the baseline characteristics of the study population included in the external validation cohort. The distribution of RAR differed markedly between the NHANES discovery cohort and the external validation cohort (Figures 2A,B). In NHANES, RAR exhibited a broad, right-skewed distribution—with substantial representation across the full spectrum, particularly in the lower range. By contrast, the validation cohort displayed a markedly narrower distribution, skewed toward higher values, with sparse representation in the lower RAR tertile or quartile.

### 3.2 Association of RAR with *Helicobacter pylori* seropositivity

Table 2 summarizes the association between RAR and *H. pylori* seropositivity across three progressively adjusted logistic regression models. In the unadjusted model, RAR was positively associated with *Helicobacter pylori* seropositivity. Each one-unit increase in RAR was linked to a 55% higher odds of seropositivity (OR = 1.55, 95% CI: 1.29–1.88, *p* < 0.0001). After adjustment for age, sex, and race/ethnicity in Model I, the association was attenuated and became non-significant (OR = 1.13, 95% CI: 0.91–1.42, *p* = 0.2718).

**Table 2:** Association between RAR and *Helicobacter pylori* seropositivity.

Exposure	Non-adjusted model	Model I	Model II
RAR	1.55 (1.29, 1.88) < 0.0001	1.13 (0.91, 1.42) 0.2718	1.01 (0.80, 1.27) 0.9380
Q1 (2.075–2.65)	Ref	Ref	Ref
Q2 (2.65–2.84)	1.58 (1.27, 1.95) < 0.0001	1.40 (1.09, 1.79) 0.0075	1.27 (0.99, 1.64) 0.0621
Q3 (2.84–3.07)	2.14 (1.73, 2.65) < 0.0001	1.62 (1.26, 2.08) 0.0001	1.49 (1.14, 1.93) 0.0031
Q4 (3.07–7.43)	1.84 (1.49, 2.28) < 0.0001	1.42 (1.10, 1.84) 0.0073	1.25 (0.95, 1.65) 0.1073
*p* for trend	<0.0001	0.0258	0.2240

Model 1 adjust for: sex, age, race. Model 2 adjust for: sex, age, race, education level, marital status, PIR, alcohol, BMI, hypertension, diabetes, coronary heart disease, stroke, smoking.

In the fully adjusted Model II, which further included socioeconomic, lifestyle, and comorbidity factors, the association remained non-significant (OR = 1.01, 95% CI: 0.80–1.27, *p* = 0.9380).

Results were directionally similar when RAR was categorized into quartiles [Q1: 2.075–2.65 (reference); Q2: 2.65–2.84; Q3: 2.84–3.07; Q4: 3.07–7.43]. In unadjusted analyses, higher quartiles were associated with greater odds of seropositivity (Q2: OR = 1.58, 95% CI: 1.27–1.95; Q3: OR = 2.14, 95% CI: 1.73–2.65; Q4: OR = 1.84, 95% CI: 1.49–2.28; all *p* < 0.0001; *p* for trend < 0.0001). After adjustment for age, sex, and race/ethnicity (Model I), the associations persisted albeit attenuated (Q2: OR = 1.40, 95% CI: 1.09–1.79; *p* = 0.0075; Q3: OR = 1.62, 95% CI: 1.26–2.08; *p* = 0.0001; Q4: OR = 1.42, 95% CI: 1.10–1.84; *p* = 0.0073; *p* for trend = 0.0258). In the fully adjusted Model II, the association was further attenuated: the Q3 category remained associated with higher odds (OR = 1.49, 95% CI: 1.14–1.93; *p* = 0.0031), whereas Q2 showed a borderline association (OR = 1.27, 95% CI: 0.99–1.64; *p* = 0.0621) and Q4 was not statistically significant (OR = 1.25, 95% CI: 0.95–1.65; *p* = 0.1073); the overall trend across quartiles was not significant (*p* for trend = 0.2240). Collectively, these cross-sectional estimates suggest that elevated RAR co-occurs with higher *H. pylori* seropositivity in crude and partially adjusted analyses, with attenuation after broader adjustment, consistent with potential confounding by sociodemographic, behavioral, and clinical factors rather than implying causality.

#### 3.2.1 Sensitivity analyses

We performed a series of sensitivity analyses to assess the robustness of the association between RAR and *H. pylori* seropositivity. In the primary adjusted model, a one–standard-deviation increase in RAR was associated with higher odds of *H. pylori* seropositivity (OR≈1.10–1.12, 95% CI approximately 1.02–1.22, Supplementary Table 3; Supplementary Figure 1). The magnitude and direction of the association were preserved across multiple perturbations: after excluding participants with possible acute inflammation (CRP > 10 mg/dL); after removing individuals with markedly elevated liver enzymes (ALT/AST/GGT ≥ 95th percentile); and after trimming extreme RAR values (1st–99th percentiles). Using a log-transformed exposure [ln(RAR)] yielded comparable estimates. When RAR was modeled in quartiles (Q1 as reference), the odds of seropositivity increased across categories, with the highest quartile showing elevated risk relative to Q1, and an overall p-for-trend = 0.016, indicating a monotonic dose–response relationship. Collectively, these results suggest that the observed association between RAR and *H. pylori* seropositivity is robust to alternative specifications and sample restrictions.

After excluding participants with clinical anemia (hemoglobin < 13 g/dL in males and < 12 g/dL in females), the association between RAR and *Helicobacter pylori* (*H. pylori*) seropositivity attenuated (Supplementary Figure 2). In the fully adjusted model, only the third quartile group retained statistical significance, while the highest quartile group exhibited a borderline association, with an overall modest trend (Supplementary Table 4).

### 3.3 Restricted cubic spline regression analysis between RAR and *Helicobacter pylori* seropositivity

**Figure 3:**
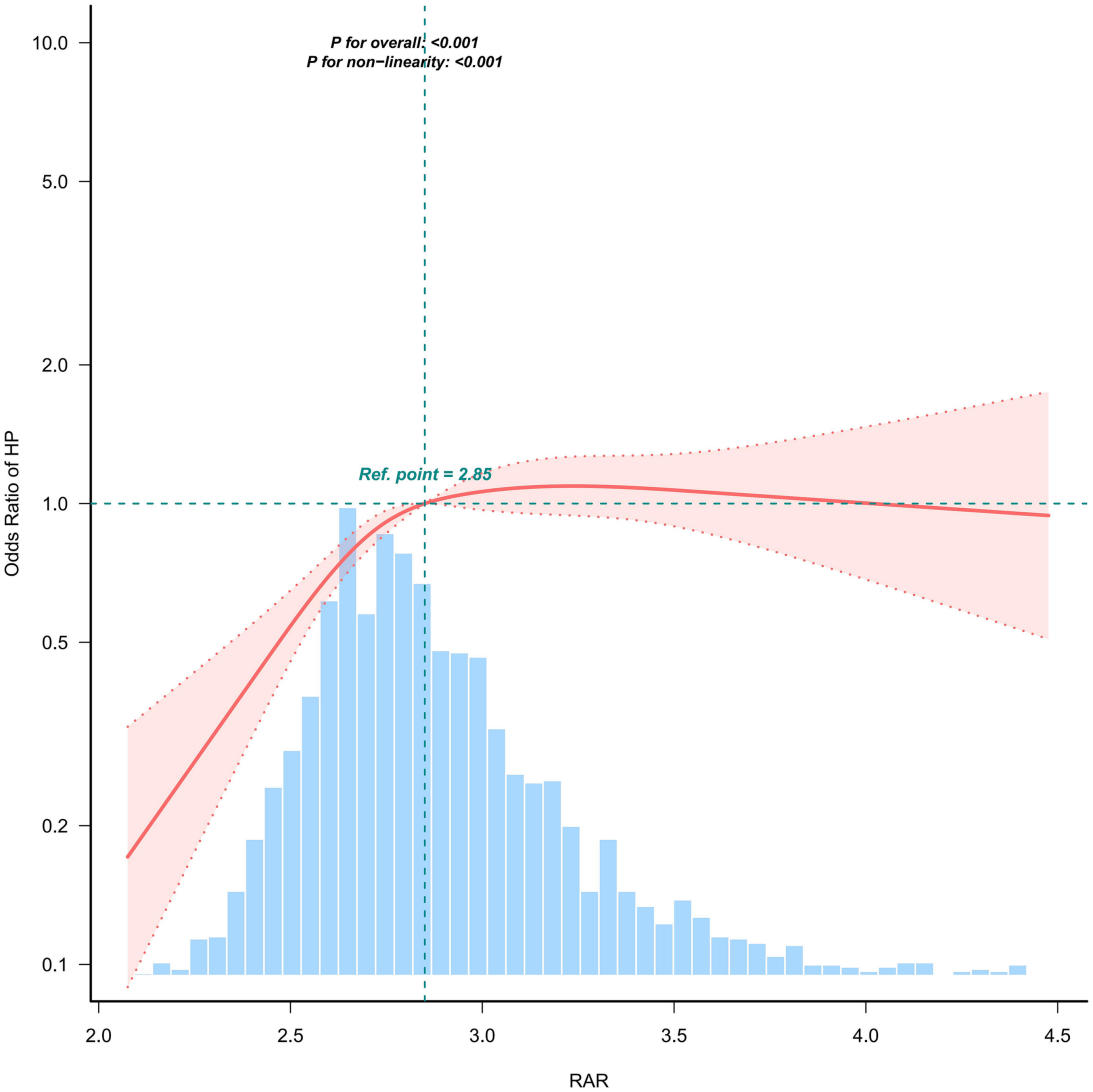
Weighted association between RAR and *Helicobacter pylori* seropositivity analyzed via restricted cubic spline model (NHANES dataset).

In the NHANES dataset, restricted cubic spline regression analysis revealed a significant nonlinear association between RAR and *Helicobacter pylori* (Hp) seropositivity (overall test *p* < 0.001; nonlinearity test *p* < 0.001). Specifically, the curve exhibited a rapid upward trend when RAR < 2.85, followed by a plateau, suggesting that RAR = 2.85 may represent the effect inflection point (Figure 3).

However, this association pattern demonstrated notable sensitivity to analytical assumptions. When the analysis was restricted to the RAR range overlapping with the external validation cohort (Supplementary Figure 3), neither the overall association nor the non-linear component attained statistical significance (overall *p* = 0.589; non-linearity *p* = 0.391). Sensitivity analyses—varying spline specifications (e.g., number and placement of knots) and excluding extreme RAR values (lowest and highest 1st percentiles)—produced qualitatively consistent dose–response curves in NHANES. Nevertheless, the estimated association remained modest in magnitude, and confidence intervals widened substantially at higher RAR levels, reflecting increased estimation uncertainty (Supplementary Figures 4A,B).

**Figure 4:**
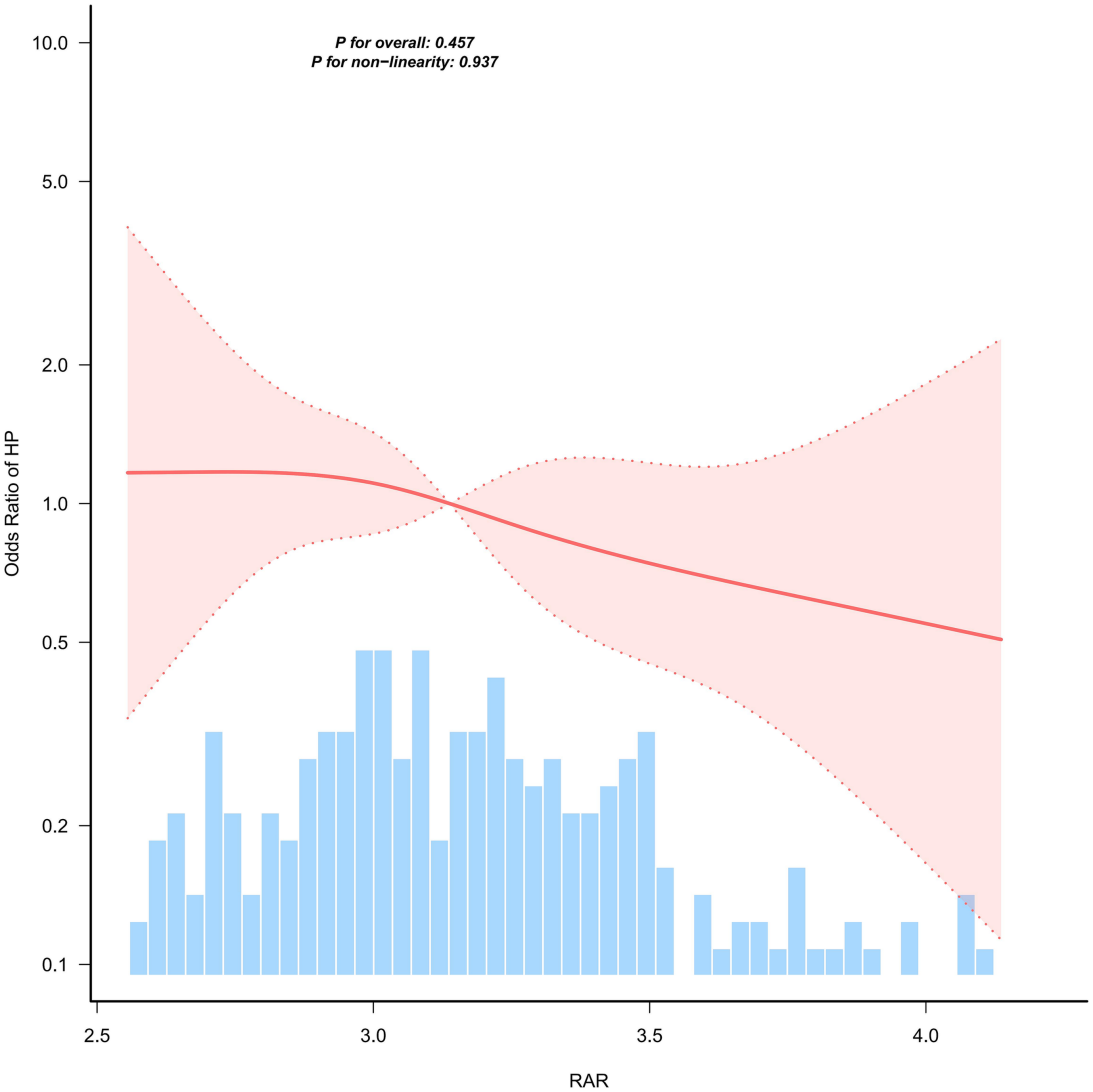
Restricted cubic spline model analysis of the association between RAR and *Helicobacter pylori* seropositivity (external validation cohort).

In the external validation cohort, RCS analysis failed to detect a significant nonlinear relationship (overall test *p* = 0.457; nonlinearity test *p* = 0.937). Within the observable exposure range, the association between RAR and Hp seropositivity was approximately linear (Figure 4).

The discrepancy in results between the two cohorts may be partially attributed to differences in the distribution of RAR: RAR values in the validation cohort were predominantly concentrated after the inflection point (RAR = 2.85), with relatively insufficient sample size in the low-RAR range. This may have compromised the statistical power to detect the nonlinear characteristics of the “steep increase segment” at low exposure levels observed in the main cohort.

**Figure 5:**
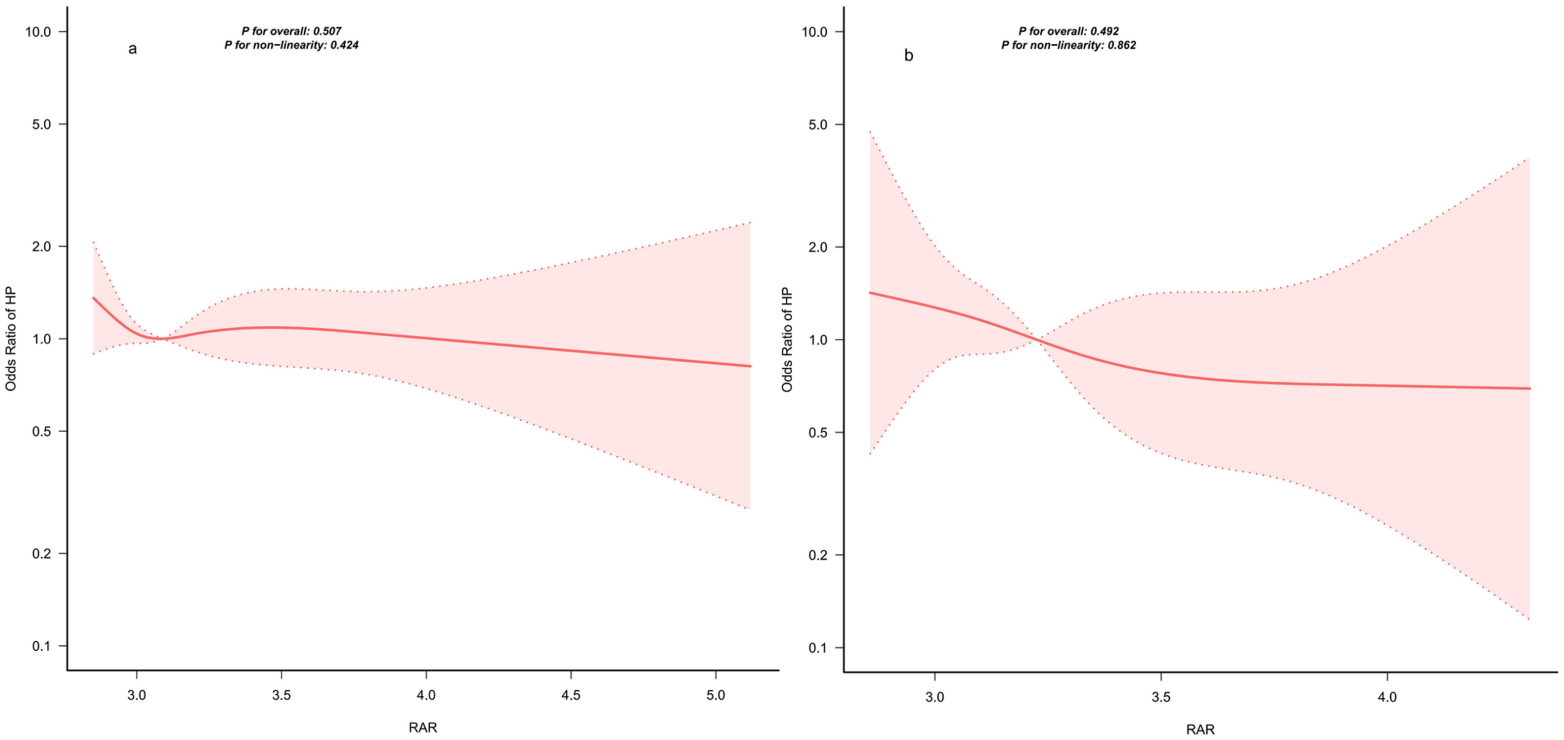
The exposure-response relationship between the two cohorts within the post-inflection point exposure range **(a)**: NHANES database, **(b)**: external validation cohort.

To further evaluate whether the between-cohort differences in findings were attributable to disparities in exposure distribution ranges, we performed a restricted analysis (inclusion criterion: RAR ≥ 2.85) on both cohorts. Within this exposure range, the restricted cubic spline (RCS) curves of both cohorts exhibited an approximately linear downward trend, with neither the overall association nor the nonlinearity test achieving statistical significance (NHANES: overall test *p* = 0.507, nonlinearity test *p* = 0.424; validation cohort: overall test *p* = 0.492, nonlinearity test *p* = 0.862) (Figure 5). These findings indicate that the nonlinear association observed in the primary cohort was primarily driven by the low-RAR interval, for which the hospital-based validation cohort had an insufficient sample size.

Exploratory analysis using two-piecewise logistic regression revealed a nonlinear relationship between RAR and *Helicobacter pylori* seropositivity, with an identified inflection point at a RAR value of 2.85. When RAR was below this threshold, each one-unit increase was significantly associated with higher odds of seropositivity, yielding an odds ratio of 3.59 and a 95% confidence interval from 1.76 to 7.31 (*p* = 0.0004). In contrast, RAR values above 2.85 were linked to decreased odds of seropositivity, with an odds ratio of 0.73 and a 95% confidence interval between 0.55 and 0.97 (*p* = 0.0318). The two-piecewise model provided a significantly better fit compared to the linear model, as indicated by the likelihood ratio test (*p* < 0.001). Consistent with this threshold effect, the fully adjusted linear model showed no significant overall association, with an odds ratio of 1.01 and a 95% confidence interval of 0.80 to 1.27 (*p* = 0.9380), further supporting a nonlinear pattern in the relationship. Given the cross-sectional design, the results are descriptive and should not be interpreted as causal (Table 3).

**Table 3:** Nonlinearity addressing of RAR and *Helicobacter pylori* seropositivity.

*Helicobacter pylori* seropositivity	OR (95% CI), *p*-value
RAR	
Fitting model by standard logistic regression	1.01 (0.80, 1.27) 0.9380
Fitting model by two-piecewise logistic regression	
Inflection point	2.85
<2.85	3.59 (1.76, 7.31) 0.0004
>2.85	0.73 (0.55, 0.97) 0.0318
*p* for log likely ratio test	<0.001

Adjusted for all covariates presented in Table 2. CI, Confidence interval; OR, Odds ratio; RAR, Red blood cell distribution width-to-albumin ratio.

### 3.4 Subgroup analyses

**Figure 6:**
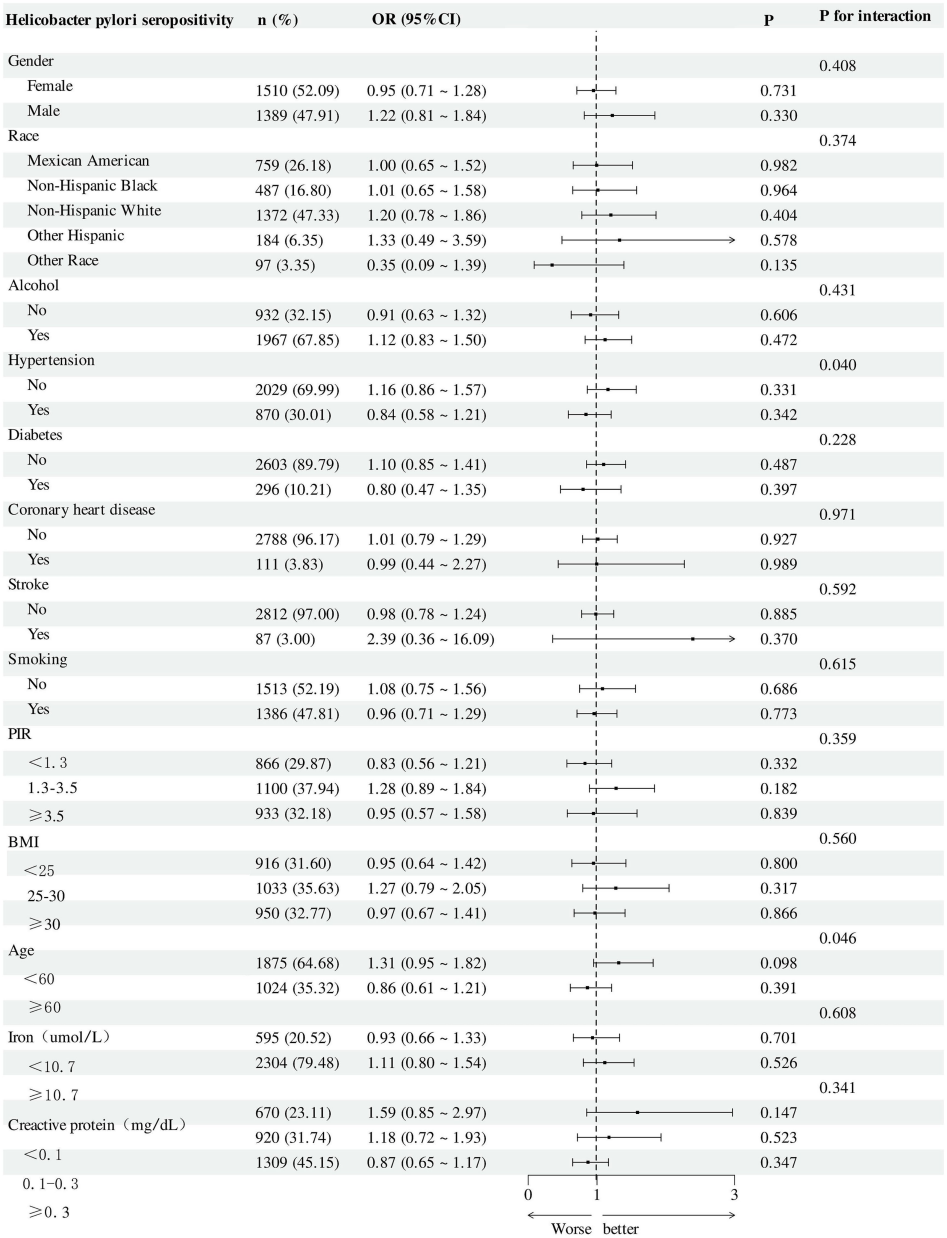
Subgroup analysis for the association between RAR and *H. pylori* seropositivity.

Figure 6 presents cross-sectional subgroup analyses of the association between RAR and the odds of *H. pylori* seropositivity. Across strata, adjusted odds ratios generally centered on the null. By sex, no clear association was observed in females (OR = 0.95, 95% CI: 0.71–1.28; *p* = 0.731) or males (OR = 1.22, 95% CI: 0.81–1.84; *p* = 0.330), and the interaction test was not significant (*p* for interaction = 0.408). Stratified by race/ethnicity, estimates ranged from 0.35 to 1.33—Mexican American: 1.00 (0.65–1.52), non-Hispanic Black: 1.01 (0.65–1.58), non-Hispanic White: 1.20 (0.78–1.86), other Hispanic: 1.33 (0.49–3.59), other race: 0.35 (0.09–1.39)—with no evidence of heterogeneity (*p* for interaction = 0.374). Similar null patterns were seen across alcohol use (*p* for interaction = 0.431), diabetes (0.228), coronary heart disease (0.971), stroke (0.592), smoking (0.615), PIR (0.359), BMI (0.560), iron (0.608), and C-reactive protein (0.341).

Two strata suggested heterogeneity of the cross-sectional association. First, hypertension showed a statistically significant interaction (*p* for interaction = 0.040): the association tended to be positive among participants without hypertension (OR = 1.16, 95% CI: 0.86–1.57; *p* = 0.331) and attenuated among those with hypertension (OR = 0.84, 95% CI: 0.58–1.21; *p* = 0.342). Second, age indicated potential heterogeneity (*p* for interaction = 0.046): estimates were higher among adults <60 years (OR = 1.31, 95% CI: 0.95–1.82; *p* = 0.096) than among those ≥60 years (OR = 0.86, 95% CI: 0.61–1.21; *p* = 0.391). The estimate among participants with prior stroke was imprecise (OR = 2.39, 95% CI: 0.36–16.09), reflecting small numbers.

Taken together, these cross-sectional findings describe largely null associations between RAR and the prevalence odds of *H. pylori* seropositivity across most subgroups, with possible variability by hypertension status and age. Given the multiple comparisons and wide confidence intervals in smaller strata, these results should be interpreted as exploratory and hypothesis-generating rather than inferential about causation.

## 4 Discussion

In this cross-sectional NHANES analysis (1999–2000), we observed that RAR related to *Helicobacter pylori* seropositivity in a nonlinear fashion. Restricted cubic spline models showed a significant overall association (*p* = 0.001) with clear nonlinearity (*p* < 0.001), yielding an inverted-U curve that peaked around RAR ≈ 3.0. Two-piecewise logistic regression identified an inflection at RAR = 2.85: below this threshold, higher RAR was associated with greater odds of seropositivity (OR = 3.59; 95% CI: 1.76–7.31), whereas above 2.85, higher RAR related to lower odds (OR = 0.73; 95% CI: 0.55–0.97). In categorical analyses, the Q3 category remained positively associated with seropositivity after full adjustment (OR = 1.49; 95% CI: 1.14–1.93), whereas Q4 was not significant and the linear trend across quartiles was null, consistent with nonlinearity. Subgroup findings were largely null across sex and race/ethnicity, with suggestive heterogeneity by age and hypertension. Collectively, these results indicate a threshold-like, nonmonotonic association between RAR and *H. pylori* serostatus in a nationally representative U.S. sample, without implying causality.

While the present study observed a non-linear association between RAR and *Helicobacter pylori* (HP) seropositivity in the primary NHANES cohort, and identified a potential inflection point at RAR = 2.85, no significant non-linearity was replicated in the hospital-based validation cohort. This discrepancy may be attributable to multiple factors. Firstly, the two cohorts exhibited distinct RAR distributions: RAR values in the validation cohort were predominantly concentrated in the post-inflection point range, with insufficient sample size in the low-RAR interval. This restricted the exposure range, reducing the statistical power of the spline model to detect curvature and resulting in an approximately linear association pattern. Secondly, the validation cohort had a relatively small sample size (*n* = 250), which may have been underpowered to capture weak non-linear patterns. Thirdly, the divergent population sources (nationally representative population vs. hospital-based patient population) may have introduced selection bias and spectrum effects, leading to heterogeneity in effect estimates. Fourthly, methodological heterogeneity across centers—including differences in testing platforms for RDW and albumin, as well as variations in HP serological reagents and positive cut-off thresholds—coupled with the long study timeframe, may have contributed to inconsistent results. Future multi-center studies with larger sample sizes and broader RAR coverage are warranted to further validate this non-linear association and its threshold effect. Notably, after restricting both cohorts to RAR ≥ 2.85, the restricted cubic spline (RCS) curves of the two cohorts showed more consistent local profiles, both displaying an approximately linear downward trend with no statistical significance in either the overall association or non-linearity tests. These sensitivity analysis findings support our interpretation: the failure to replicate the non-linear association in the validation cohort is primarily due to restricted exposure range and insufficient sample size in the low-RAR interval, rather than a direct refutation of the primary cohort findings.

This cross-sectional study is the first to investigate the association between RAR and *Helicobacter pylori* seropositivity using NHANES data. While direct evidence is limited, previous studies have identified RAR as a marker of systemic inflammation and metabolic dysfunction, supporting its relevance in *H. pylori*–related pathology. Prior research has reported that elevated RAR levels are associated with adverse outcomes in diseases such as acute respiratory failure, diabetic ketoacidosis, sepsis, pulmonary embolism, and stroke, all of which involve acute or chronic inflammation (23–26). Some of these conditions may share pathophysiological pathways with gastrointestinal infections such as *H. pylori*, including systemic inflammation and metabolic dysregulation. Regarding *H. pylori*, several studies have shown associations between the infection and metabolic or inflammatory markers. For example, Xu et al. identified a positive association between *H. pylori* seropositivity and the HbA1c/HDL-C ratio in NHANES data, suggesting a potential metabolic link (18). Similarly, Xie et al. found that *H. pylori* infection was associated with elevated triglyceride levels, possibly reflecting a state of low-grade inflammation (17). Our study found that higher RAR was significantly associated with *H. pylori* seropositivity in unadjusted models, but this association disappeared after full adjustment. Non-linear analyses suggested a possible inverted U-shaped relationship, potentially reflecting complex interactions with inflammation, nutrition, or other unmeasured factors. Notably, findings from other populations have differed. For example, a study by Vayá et al. conducted among healthy Japanese adults did not observe a significant association between RDW and *H. pylori* infection (27). Such discrepancies may be explained by differences in population characteristics (e.g., overall health status, dietary patterns), lower infection prevalence, smaller sample size, or differences in diagnostic methods (e.g., stool antigen vs. serum IgG testing), all of which could influence the sensitivity and specificity of *H. pylori* detection. Together, these findings suggest that RAR may reflect inflammation- and nutrition-related alterations associated with *H. pylori* infection, supporting its potential utility as a non-specific biomarker in gastrointestinal or metabolic contexts.

To gain a deeper understanding of the relationship between RAR and *Helicobacter pylori* seropositivity, multiple analytical approaches were applied. These included multivariable logistic regression, subgroup analyses stratified by key variables, and restricted cubic spline modeling to assess potential nonlinearity. Although both the unadjusted and partially adjusted models indicated a significant positive association, this relationship diminished and lost statistical significance after full adjustment. This attenuation suggests that sociodemographic and clinical factors may have confounded the observed association. Nonlinear analyses revealed a threshold-like, inverted-U association between RAR and *H. pylori* seropositivity, with an inflection point around RAR = 2.85. Subgroup analyses showed largely null results, though exploratory evidence of heterogeneity by age and hypertension status was observed. These findings highlight the complexity of the RAR–*H. pylori* relationship and the possibility that inflammatory or nutritional disruptions may have context-specific effects. Given the cross-sectional nature of this study, causality cannot be established. Longitudinal investigations are warranted to determine whether RAR represents a modifiable marker of infection risk or reflects downstream consequences of chronic *H. pylori* exposure.

Recent studies suggest that RAR may reflect a combination of systemic inflammation, nutritional status, and oxidative stress—factors that are also involved in the pathophysiology of *Helicobacter pylori* infection. RDW tends to rise in response to inflammatory cytokines and oxidative stress, while albumin levels often decrease in chronic inflammatory or malnourished states. As such, a higher RAR could indicate an unfavorable physiological environment that either predisposes individuals to *H. pylori* infection or results from it (12, 25, 28).

*Helicobacter pylori* is known to trigger a chronic inflammatory response in the gastric mucosa, leading to the release of interleukin-6 and tumor necrosis factor-alpha, which can interfere with red blood cell production and reduce albumin synthesis. Over time, this may contribute to higher RDW and lower albumin levels, thereby elevating RAR in infected individuals (25). At the same time, chronic gastritis and impaired nutrient absorption—both associated with *H. pylori*—may lead to deficiencies in iron, vitamin B12, or protein, further affecting RAR-related parameters (12).

It is also important to consider the possibility of reverse causation. Individuals with systemic inflammation or malnutrition—conditions that increase RAR—might be more vulnerable to *H. pylori* colonization or prolonged seropositivity. In this context, elevated RAR may not only reflect consequences of the infection but also signal a host environment more susceptible to persistent *H. pylori* colonization or sustained seropositivity (26, 29). Altogether, these insights point to RAR as a marker that captures the inflammatory and nutritional milieu associated with *H. pylori* seropositivity or prior exposure, rather than definitively confirmed active infection. An important consideration is that *H. pylori* status in NHANES 1999–2000 was defined by serum anti–*H. pylori* IgG positivity rather than by methods that identify current infection, such as the urea breath test, stool antigen testing, or biopsy-based assessment. Because IgG antibodies may persist long after prior exposure or successful eradication, seropositivity cannot be assumed to represent ongoing infection at the time of assessment. Therefore, the associations observed in this study should be interpreted as relationships between RAR and *H. pylori* serostatus/exposure history. This limitation may have introduced non-differential misclassification of the outcome and may partly explain why the observed associations were modest after full adjustment. Further studies using direct measures of active infection are needed to determine whether the nonlinear pattern identified here also applies to current *H. pylori* infection.

Our subgroup analyses revealed that the overall association between RAR and *H. pylori* seropositivity was largely consistent across demographic and clinical strata, with no strong interaction effects observed. However, hypertension and age appeared to modify the association modestly. Specifically, in individuals without hypertension and those under 60 years of age, higher RAR levels tended to show a positive relationship with *H. pylori* seropositivity, whereas this pattern was less evident in older adults and those with hypertension. These trends may reflect age-related changes in immune responsiveness or the influence of chronic comorbidities, which can alter inflammatory profiles and nutritional status—factors closely linked to RAR (29, 30). Notably, previous studies have identified RAR as a composite marker of systemic inflammation and nutritional decline, particularly in elderly or metabolically vulnerable populations (12, 31). Still, given the cross-sectional nature of our data and the limited statistical power in some subgroups, these findings should be considered exploratory. Further longitudinal research is needed to assess whether factors such as hypertension and age genuinely modify the link between RAR and *H. pylori* seropositivity or active infection or merely reflect residual confounding.

A further limitation concerns the temporal relevance of the NHANES 1999–2000 dataset. This cycle was selected as the most recent nationally representative survey to include both Hp serology and all laboratory parameters necessary for RAR calculation. To our knowledge, no subsequent NHANES cycle has incorporated Hp serologic testing—rendering this dataset uniquely valuable yet temporally constrained for investigating this association.

We acknowledge that dietary patterns, antibiotic exposure (including widespread use for non-gastrointestinal indications), and the epidemiology of Hp infection in the United States have shifted meaningfully over the past two decades. Consequently, the external validity of our findings to current U.S. populations is likely attenuated. Our results should therefore be interpreted not as a basis for clinical screening, but as exploratory and hypothesis-generating—identifying a biologically plausible link between systemic inflammation–nutrition status and Hp seropositivity that merits rigorous validation in contemporary, prospectively designed studies with updated exposure and outcome assessments.

### 4.1 Limitations

Several caveats warrant mention. First, NHANES employs a cross-sectional design, precluding inference about temporal sequence and leaving reverse causation as a key interpretive limitation. Chronic Hp infection may itself drive systemic inflammation, nutritional deficits, and hematologic perturbations—thereby elevating RAR—rather than elevated RAR predisposing to infection. Consequently, the observed associations are best interpreted as descriptive of physiological alterations accompanying Hp seropositivity, not as evidence of causal risk effects. Second, Hp status was defined by serum IgG seropositivity—a marker of cumulative exposure or prior infection—not active, contemporaneous colonization. Because IgG antibodies persist after eradication or remote infection, this definition risks outcome misclassification and weakens alignment between serostatus and current inflammatory or nutritional physiology. Moreover, the dataset lacked data on bacterial load, virulence markers (e.g., CagA, VacA), and history of prior eradication therapy. Third, residual confounding remains plausible. Although we adjusted for major demographic, socioeconomic, behavioral, anthropometric, and clinical covariates available in NHANES, Hp seropositivity is shaped by additional determinants—including childhood living conditions, household crowding, sanitation infrastructure, healthcare access, prior treatment, reinfection risk, and strain-specific virulence—most of which were unmeasured. Thus, even our fully adjusted model cannot rule out residual confounding, and findings must be regarded as associative, not causal. Fourth, the external validation cohort exhibited limited representation of low RAR values and a narrower exposure distribution than NHANES. This constrained its capacity to replicate the non-linear association observed in the primary analysis. Consistently, when the NHANES analysis was restricted to the RAR range overlapping with the validation cohort, the association lost statistical significance—indicating that the observed non-linearity is sensitive to exposure distribution and may reflect extrapolation beyond the empirically supported range. Fifth, RAR was derived from single-point measurements of RDW and albumin—both subject to biological and technical variability. RDW may be influenced by iron, vitamin B12, or folate deficiency; chronic liver or kidney disease; hydration status; acute illness; and medications (e.g., proton pump inhibitors, antibiotics). Albumin is similarly affected by hepatic synthesis, renal loss, inflammation, and nutritional intake. Despite multivariable adjustment, unmeasured or imperfectly captured sources of variation likely persist. Sixth, although all NHANES analyses accounted for the complex survey design—including Mobile Examination Center (MEC) examination weights, strata, and primary sampling units—survey weighting mitigates only sampling-related bias. It cannot address bias arising from unmeasured confounders, systematic measurement error, or outcome misclassification. Seventh, methodological and temporal limitations apply: laboratory assays used in NHANES 1999–2000 differ from contemporary standards, and the survey cycle predates major shifts in U.S. dietary patterns, antibiotic utilization, and Hp epidemiology (e.g., declining prevalence, aging seropositive cohorts). These factors limit generalizability to current clinical practice and non-U.S. populations. Eighth, missing data led to substantial sample attrition. Among adults, exclusions for missing RAR components, unavailable Hp serology, or incomplete covariate data yielded a complete-case analytic sample of 2,899 participants (43.2% of the eligible adult population). If missingness correlated with sociodemographic disadvantage, comorbidities, nutritional or inflammatory status, or Hp serostatus itself, selection bias may have arisen. Furthermore, complete-case analysis reduces statistical power and may destabilize spline-based estimates of non-linear associations. Future studies should employ robust missing-data methods—such as multiple imputation or inverse probability weighting—to assess sensitivity. Finally, the absence of gastric histopathology, gut microbiome profiling, and comprehensive inflammatory or dietary biomarkers limits mechanistic insight. Prospective studies with repeated exposure and outcome assessments—and deeper molecular and clinical phenotyping—are needed to validate and extend these observations.

## 5 Conclusion

In this nationally representative cross-sectional study, we observed a statistically significant non-linear association between RAR and *H. pylori* seropositivity in NHANES; however, this association lacked robustness across prespecified analytical conditions. It attenuated after multivariable adjustment and further diminished following exclusion of participants with clinical anemia—suggesting that hematologic and nutritional confounding, rather than a direct biological effect of RAR, may partially underlie the observed signal. Critically, the association failed to replicate in the external validation cohort and became non-significant when analyses were restricted to the RAR range overlapping with that cohort—highlighting sensitivity to both population heterogeneity and exposure distribution. Collectively, these findings indicate that RAR likely serves as a composite marker reflecting the systemic inflammation–nutrition milieu associated with *H. pylori* seropositivity or prior exposure, rather than a specific, independent predictor. Consequently, RAR does not meet criteria for a reliable or clinically actionable standalone biomarker. Prospective longitudinal studies and mechanistic investigations are warranted to clarify causal directionality and assess whether RAR—or its constituent components—holds utility in risk stratification or pathophysiological modeling.

## Data Availability

The original contributions presented in the study are included in the article/Supplementary material, further inquiries can be directed to the corresponding author/s.

## Footnotes

1
https://www.cdc.gov/nchs/nhanes/

## Supplementary material

SUPPLEMENTARY FIGURE 1 Forest plot of sensitivity analyses for the association between RAR and *H. pylori* seropositivity.

SUPPLEMENTARY FIGURE 2 Restricted cubic spline (RCS) analysis after exclusion of participants with clinical anemia.

SUPPLEMENTARY FIGURE 3 Restricted cubic spline (RCS) analysis restricted to overlapping RAR range.

SUPPLEMENTARY FIGURE 4 Sensitivity analyses of spline model specification.

## Glossary

ALT: Alanine aminotransferase
AST: Aspartate aminotransferase
AUC: Area under the curve
BMI: Body mass index
BCP: Bromocresol purple
CDC: Centers for Disease Control and Prevention
CI: Confidence interval
CRP: C-reactive protein
ELISA: Enzyme-linked immunosorbent assay
GGT: Gamma-glutamyltransferase
*H. pylori*: *Helicobacter pylori*
IgG: Immunoglobulin G
NAFLD: Non-alcoholic fatty liver disease
NHANES: National Health and Nutrition Examination Survey
NCHS: National Center for Health Statistics
OR: Odds ratio
PIR: Poverty to income ratio
PSU: Primary sampling unit
RAR: Red blood cell distribution width-to-albumin ratio
RDW: Red blood cell distribution width
RCS: Restricted cubic spline
ROC: Receiver operating characteristic
SD: Standard deviation
SE: Standard error
VIF: Variance inflation factor
WHO: World Health Organization

## References

[1] Malfertheiner P Camargo MC El-Omar E Liou JM Peek R Schulz C . *Helicobacter pylori* infection. Nat Rev Dis Prim. (2023) 9:19. doi: 10.1038/s41572-023-00431-8, 37081005 PMC11558793

[2] Umar Z Tang JW Marshall BJ Tay ACY Wang L. Rapid diagnosis and precision treatment of *Helicobacter pylori* infection in clinical settings. Crit Rev Microbiol. (2025) 51:369–98. doi: 10.1080/1040841x.2024.2364194, 38910506

[3] Xu W Xu L Xu C. Relationship between *Helicobacter pylori* infection and gastrointestinal microecology. Front Cell Infect Microbiol. (2022) 12:938608. doi: 10.3389/fcimb.2022.938608, 36061875 PMC9433739

[4] O'Connor A O'Morain CA Ford AC. Population screening and treatment of Helicobacter pylori infection. Nat Rev Gastroenterol Hepatol. (2017) 14:230–40. doi: 10.1038/nrgastro.2016.195 28053340

[5] Flores-Treviño S Mendoza-Olazarán S Bocanegra-Ibarias P Maldonado-Garza HJ Garza-González E. *Helicobacter pylori* drug resistance: therapy changes and challenges. Expert Rev Gastroenterol Hepatol. (2018) 12:819–27. doi: 10.1080/17474124.2018.1496017 29976092

[6] Roszczenko-Jasińska P Wojtyś MI Jagusztyn-Krynicka EK. *Helicobacter pylori* treatment in the post-antibiotics era-searching for new drug targets. Appl Microbiol Biotechnol. (2020) 104:9891–905. doi: 10.1007/s00253-020-10945-w, 33052519 PMC7666284

[7] Ghazanfar H Javed N Reina R Thartori O Ghazanfar A Patel H. Advances in diagnostic modalities for *Helicobacter pylori* infection. Life (Basel, Switzerland). (2024) 14:1170. doi: 10.3390/life14091170, 39337953 PMC11432972

[8] Ishida Y Suzuki K Taki K Niwa T Kurotsuchi S Ando H . Significant association between *Helicobacter pylori* infection and serum C-reactive protein. Int J Med Sci. (2008) 5:224–9. doi: 10.7150/ijms.5.224, 18695743 PMC2500148

[9] Haile K Timerga A. Evaluation of hematological parameters of *Helicobacter pylori*-infected adult patients at southern Ethiopia: a comparative cross-sectional study. J Blood Med. (2021) 12:77–84. doi: 10.2147/jbm.S294958, 33654446 PMC7910148

[10] Liu H Qin Y Yang J Huang G Wei X Wang L . *Helicobacter pylori* infection as a risk factor for abnormal serum protein levels in general population of China. J Inflamm Res. (2022) 15:2009–17. doi: 10.2147/jir.S355446, 35370414 PMC8968220

[11] Acehan F Aslan M Demir MS Koç Ş Dügeroğlu B Kalkan C . The red cell distribution width-to-albumin ratio: a simple index has high predictive accuracy for clinical outcomes in patients with acute pancreatitis. Pancreatology. (2024) 24:232–40. doi: 10.1016/j.pan.2023.12.015, 38184456

[12] Luo J Zhu P Zhou S. Association between the red blood cell distribution width-to-albumin ratio and risk of colorectal and gastric cancers: a cross-sectional study using NHANES 2005-2018. BMC Gastroenterol. (2025) 25:316. doi: 10.1186/s12876-025-03871-6, 40301760 PMC12042377

[13] Yang H Liang L Liu Y Wang X Li J Du X . Prognostic significance of red cell distribution width to albumin ratio in ICU patients with non-alcoholic fatty liver disease: a retrospective analysis. BMC Gastroenterol. (2025) 25:485. doi: 10.1186/s12876-025-04019-2, 40597704 PMC12211306

[14] Zhou Y Zhao L Tang Y Qian S. Association between red blood cell distribution width-to-albumin ratio and depression: a cross-sectional analysis among US adults, 2011-2018. BMC Psychiatry. (2025) 25:464. doi: 10.1186/s12888-025-06907-z, 40335911 PMC12060335

[15] Hao M Jiang S Tang J Li X Wang S Li Y . Ratio of red blood cell distribution width to albumin level and risk of mortality. JAMA Netw Open. (2024) 7:e2413213. doi: 10.1001/jamanetworkopen.2024.13213, 38805227 PMC11134218

[16] Sualeheen A Tan SY Daly RM Georgousopoulou E Roberts SK George ES. Higher diet quality is associated with a lower prevalence of MASLD and adverse health outcomes: insights from NHANES 2005 to 2020. Eur J Nutr. (2025) 64:289. doi: 10.1007/s00394-025-03809-4, 41051618 PMC12500818

[17] Xie J Wang J Zeng R Xie Y. Association between *Helicobacter pylori* infection and triglyceride levels: a nested cross-sectional study. Front Endocrinol. (2023) 14:1220347. doi: 10.3389/fendo.2023.1220347, 37664839 PMC10468968

[18] Xu C Jiang XY Liao JM Zhao YF Hu JY Li CC . Association between *Helicobacter pylori* seropositivity and the hemoglobin A1c/high-density lipoprotein cholesterol ratio in U.S. adults: evidence from NHANES. Front Nutr. (2025) 12:15895. doi: 10.3389/fnut.2025.1589510 PMC12183076 40551731

[19] Zhao Z Ma Y Huangfu W. Red cell distribution width-to-albumin ratio and hypertension risk: age-specific threshold effects identified in the 2017-2020 NHANES U.S. adult population. BMC Cardiovasc Disord. (2025) 25:5072. doi: 10.1186/s12872-025-05072-1, 40783688 PMC12335172

[20] Zhou K Rao Z Chen I Guo Z Luo J Zhang Y. Association of overactive bladder with red cell distribution width to albumin ratio in American. Sci Rep. (2025) 15:27395. doi: 10.1038/s41598-025-13129-6, 40721625 PMC12304304

[21] Zhang L Sun H Liu Z Yang J Liu Y. Association between dietary sugar intake and depression in US adults: a cross-sectional study using data from the National Health and nutrition examination survey 2011-2018. BMC Psychiatry. (2024) 24:110. doi: 10.1186/s12888-024-05531-7, 38326834 PMC10851576

[22] Zhou X Zhao J Liu Y Sun X Li X Ren J . Association between serum potassium and Parkinson's disease in the US (NHANES 2005-2020). Front Neurosci. (2024) 18:1387266. doi: 10.3389/fnins.2024.1387266, 38784091 PMC11111918

[23] He Q Hu S Xie J Liu H Li C. The red blood cell distribution width to albumin ratio was a potential prognostic biomarker for acute respiratory failure: a retrospective study. BMC Med Inform Decis Mak. (2024) 24:253. doi: 10.1186/s12911-024-02639-4, 39272143 PMC11394933

[24] Zhou D Wang J Li X. The red blood cell distribution width-albumin ratio was a potential prognostic biomarker for diabetic ketoacidosis. Int J Gen Med. (2021) 14:5375–80. doi: 10.2147/ijgm.S327733, 34522133 PMC8434876

[25] Shan X Li Z Jiang J Li W Zhan J Dong L. Prognostic value of red blood cell distribution width to albumin ratio for predicting mortality in adult patients meeting sepsis-3 criteria in intensive care units. BMC Anesthesiol. (2024) 24:208. doi: 10.1186/s12871-024-02585-8, 38877408 PMC11177566

[26] Eyiol A Ertekin B. Association of red blood cell distribution width to albumin ratio with prognosis in stroke patients. Biomark Med. (2024) 18:311–20. doi: 10.2217/bmm-2023-0460, 38648096 PMC11218802

[27] Vayá A Sarnago A Fuster O Alis R Romagnoli M. Influence of inflammatory and lipidic parameters on red blood cell distribution width in a healthy population. Clin Hemorheol Microcirc. (2015) 59:379–85. doi: 10.3233/ch-141862, 25159489

[28] Shan X Jiang J Li W Dong L. Red blood cell distribution width to albumin ratio as a predictor of mortality in ICU patients with community acquired bacteremia. Sci Rep. (2024) 14:28596. doi: 10.1038/s41598-024-80017-w, 39562694 PMC11576904

[29] Yu B Li M Yu Z Zhang H Feng X Gao A . Red blood cell distribution width to albumin ratio (RAR) is associated with low cognitive performance in American older adults: NHANES 2011-2014. BMC Geriatr. (2025) 25:157. doi: 10.1186/s12877-025-05800-4, 40055657 PMC11887108

[30] Lin J Zhang L Deng S Feng B Liu L Fan G. Ratio of red blood cell distribution width to albumin: a predictive biomarker of in-hospital mortality in heart failure patients. Acta Cardiol. (2025) 80:376–86. doi: 10.1080/00015385.2025.2491151, 40223642

[31] Chen J Zhang D Zhou D Dai Z Wang J. Association between red cell distribution width/serum albumin ratio and diabetic kidney disease. J Diabetes. (2024) 16:e13575. doi: 10.1111/1753-0407.13575, 38923843 PMC11200132

