# Supplementary figures and images for: The association between red blood cell distribution width-to-albumin ratio and Helicobacter pylori seropositivity in U.S. adults: an observational study from NHANES 1999–2000 with external validation
Source: Front Nutr. 2026 May 8;13:1781850. doi: 10.3389/fnut.2026.1781850 (PMC13220124; doi:10.3389/fnut.2026.1781850)

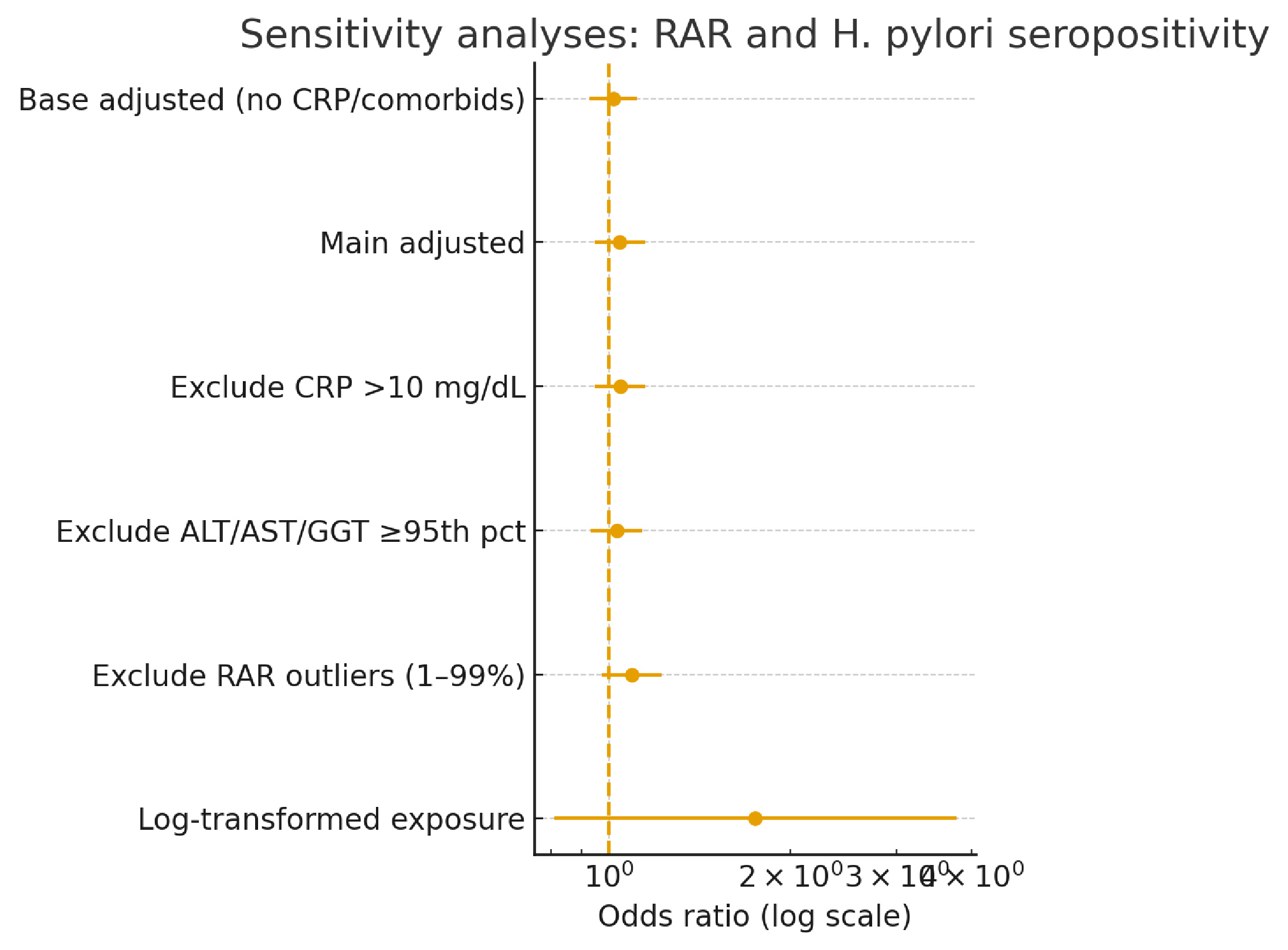

Supplement: SUPPLEMENTARY FIGURE 1 — Forest plot of sensitivity analyses for the association between RAR and H. pylori seropositivity. [file Image_1.JPEG]

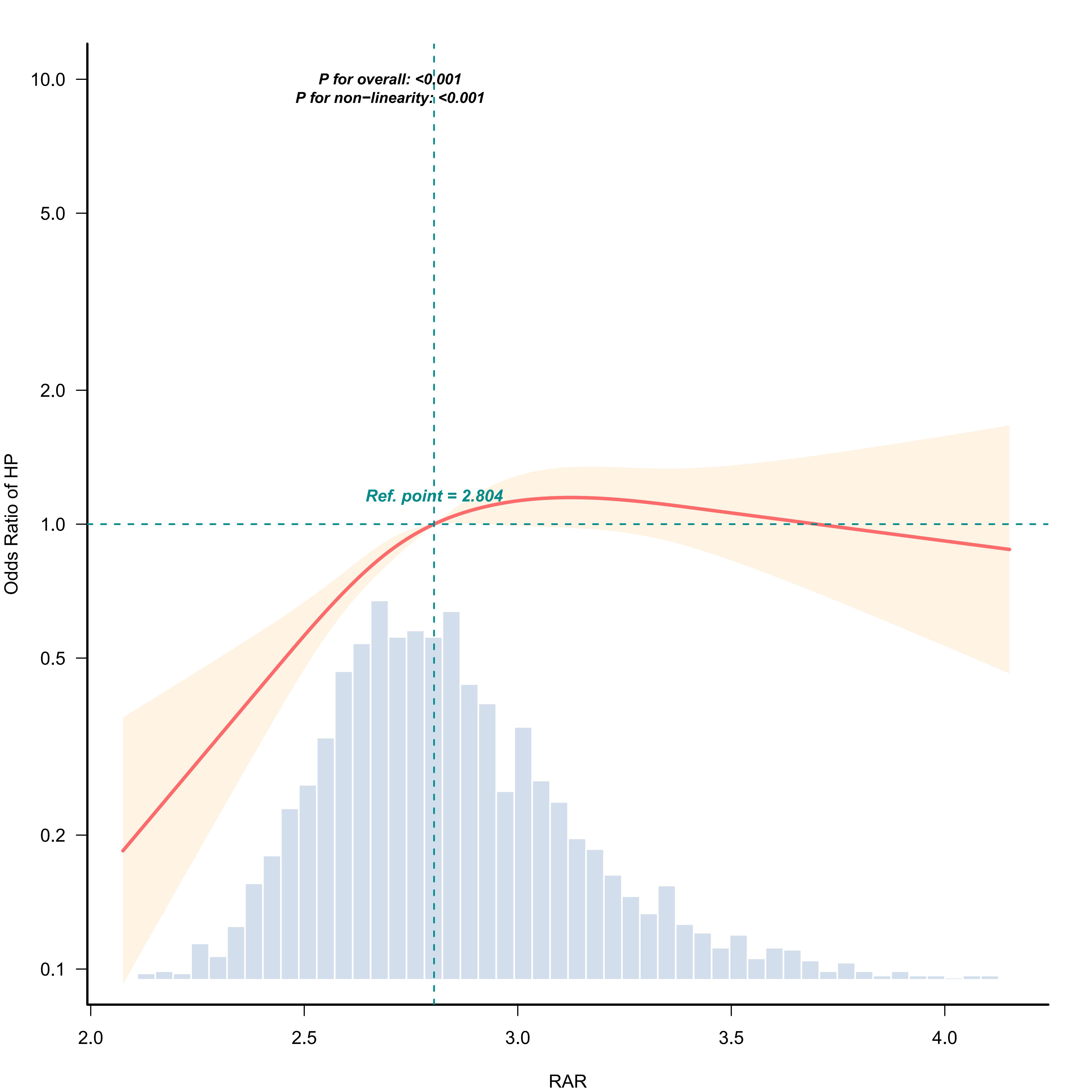

Supplement: SUPPLEMENTARY FIGURE 2 — Restricted cubic spline (RCS) analysis after exclusion of participants with clinical anemia. [file Image_2.JPEG]

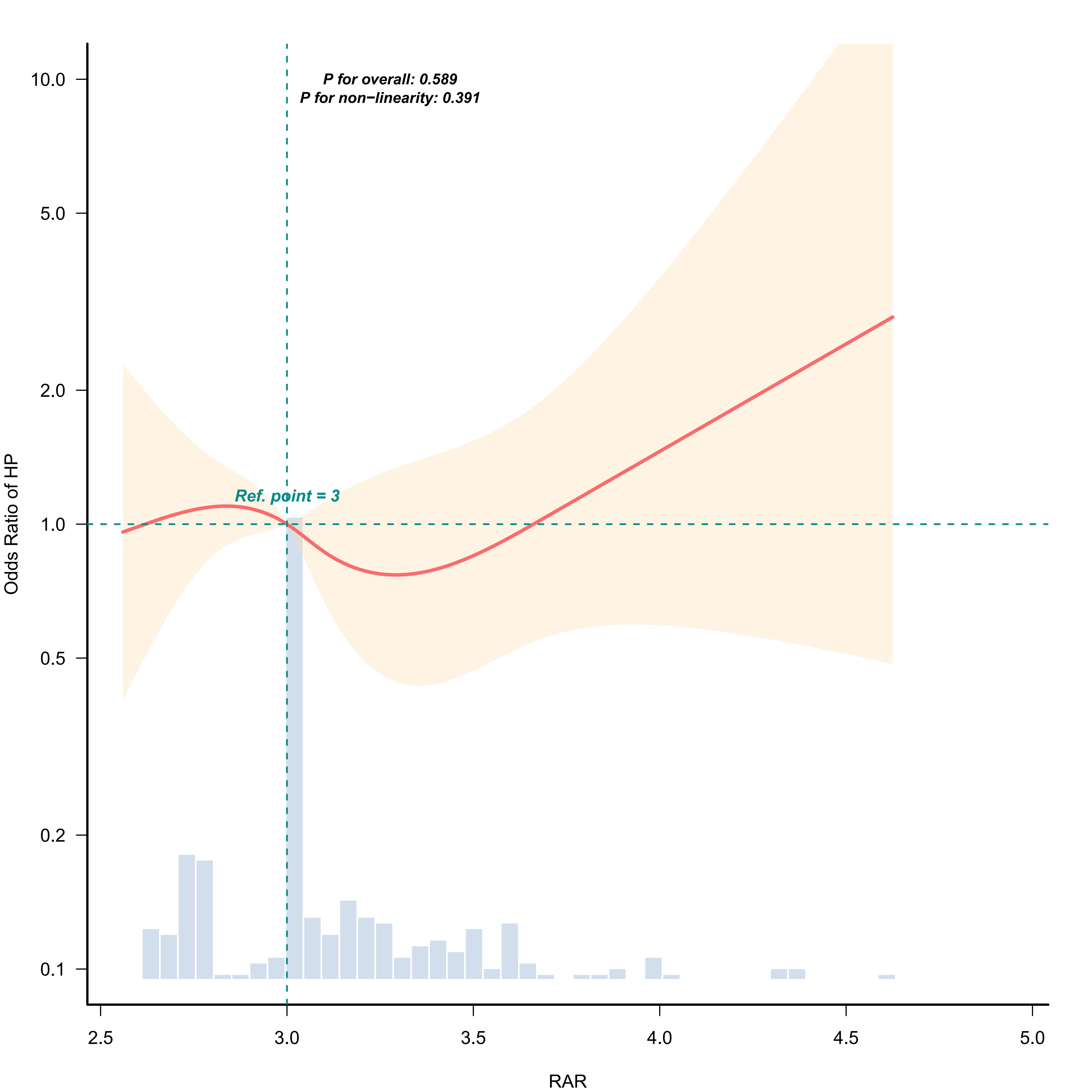

Supplement: SUPPLEMENTARY FIGURE 3 — Restricted cubic spline (RCS) analysis restricted to overlapping RAR range. [file Image_3.JPEG]

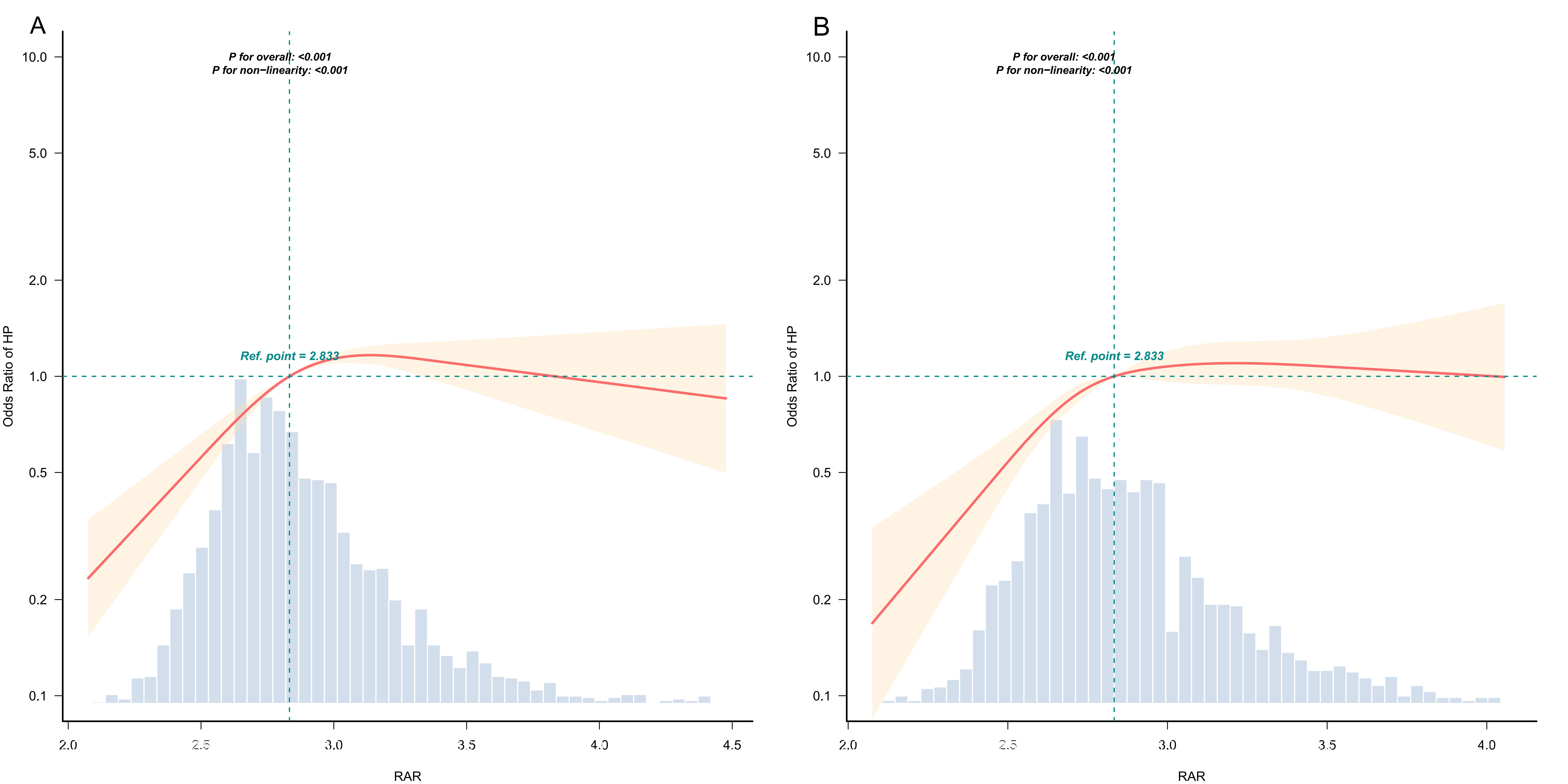

Supplement: SUPPLEMENTARY FIGURE 4 — Sensitivity analyses of spline model specification. [file Image_4.JPEG]
