# Supplementary material for: The association between red blood cell distribution width-to-albumin ratio and Helicobacter pylori seropositivity in U.S. adults: an observational study from NHANES 1999–2000 with external validation
Source: Front Nutr. 2026 May 8;13:1781850. doi: 10.3389/fnut.2026.1781850 (PMC13220124; doi:10.3389/fnut.2026.1781850)
Supplement: Supplementary file 5 [file Table_1.DOCX]

Supplementary Table 1. VIFs for covariates in multivariable models of the association between RAR and Helicobacter pylori seropositivity

| Variable (encoded) | VIF |
| --- | --- |
| Race_Non-Hispanic White | 1.934616551455739 |
| EducationLevel_Less than high school | 1.8014951902684462 |
| Age | 1.6326712539219765 |
| Race_Non-Hispanic Black | 1.4908492121122543 |
| PIR | 1.4292351779856132 |
| MaritalStatus_Never married | 1.2764825662176322 |
| EducationLevel_High school | 1.2747028021289954 |
| Hypertension_Yes | 1.2708017204040143 |
| MaritalStatus_Widowed/Divorced/Separated | 1.2423525009014094 |
| Alcohol_Yes | 1.2237680303152938 |
| Gender_Male | 1.198062598840234 |
| Race_Other Hispanic | 1.1891090301813598 |
| Smoking_Yes | 1.1716974072769917 |
| Race_Other Race | 1.12371346724023 |
| Diabetes_Yes | 1.1193429049654535 |
| BMI | 1.082457469666031 |
| Coronary heart disease_Yes | 1.0690650962137853 |
| Stroke_Yes | 1.0523440439798795 |

Supplementary Table 2. Baseline characteristics of participants in the external validation cohort

| **Variables** | **Total**  **(n = 250)** | **H.Pylorisero negative**  **(n = 125)** | **H.Pyloriser opositive**  **(n = 125)** | ***p*** |
| --- | --- | --- | --- | --- |
| Age, Median (IQR) | 60.0 (52.0, 68.0) | 59.0 (51.0, 66.0) | 62.0 (53.0, 69.0) | 0.028 |
| Gender, n (%) |  |  |  | 0.016 |
| Female | 135 (54.0) | 77 (61.6) | 58 (46.4) |  |
| Male | 115 (46.0) | 48 (38.4) | 67 (53.6) |  |
| Marital Status, n (%) |  |  |  | 1 |
| Unmarried | 3 ( 1.2) | 2 (1.6) | 1 (0.8) |  |
| Married | 247 (98.8) | 123 (98.4) | 124 (99.2) |  |
| Alcohol, n (%) |  |  |  | < 0.001 |
| No | 117 (46.8) | 95 (76) | 22 (17.6) |  |
| Yes | 133 (53.2) | 30 (24) | 103 (82.4) |  |
| Smoking, n (%) |  |  |  | < 0.001 |
| No | 106 (42.4) | 71 (56.8) | 35 (28) |  |
| Yes | 144 (57.6) | 54 (43.2) | 90 (72) |  |
| Cholesterol total(mmol/L), Median (IQR) | 4.8 (4.0, 5.3) | 4.7 (4.0, 5.1) | 4.8 (4.1, 5.4) | 0.174 |
| Creactiveprotein(mg/dL), Median (IQR) | 1.1 (0.5, 2.7) | 1.0 (0.5, 2.3) | 1.1 (0.5, 3.1) | 0.449 |
| ALT(U/L), Median (IQR) | 16.7 (12.0, 24.3) | 16.0 (11.5, 22.5) | 17.0 (12.6, 28.9) | 0.064 |
| Triglycerides(mmol/L), Median (IQR) | 1.5 (1.1, 2.1) | 1.5 (1.1, 2.0) | 1.6 (1.1, 2.1) | 0.594 |
| GGT(U/L), Median (IQR) | 21.4 (14.9, 35.0) | 18.4 (14.0, 29.0) | 25.7 (16.7, 41.0) | 0.001 |
| Creatinine(mg/dL), Median (IQR) | 62.5 (53.0, 74.0) | 60.6 (52.2, 71.1) | 65.8 (54.0, 77.5) | 0.105 |
| Glucose(mmol/L), Median (IQR) | 5.1 (4.5, 5.8) | 5.0 (4.4, 5.7) | 5.2 (4.6, 5.9) | 0.079 |
| Hb(g/L), Median (IQR) | 134.0 (122.0, 145.0) | 131.0 (120.0, 141.0) | 136.0 (124.0, 149.0) | 0.007 |
| AST(U/L), Median (IQR) | 20.0 (16.6, 25.0) | 19.2 (16.0, 24.0) | 20.9 (17.7, 25.9) | 0.05 |
| Hypertension, n (%) | 119 (47.6) | 47 (37.6) | 72 (57.6) | 0.002 |
| Diabetes, n (%) | 62 (24.8) | 22 (17.6) | 40 (32) | 0.008 |
| Coronary.heart.disease, n (%) | 25 (10.0) | 12 (9.6) | 13 (10.4) | 0.833 |
| Stroke, n (%) | 25 (10.0) | 10 (8) | 15 (12) | 0.292 |

Supplementary Table 3. Sensitivity analyses of the association between RAR and H. pylori seropositivity under alternative model specifications and sample restrictions

| Analysis | Exposure | OR (95% CI) | P |
| --- | --- | --- | --- |
| Base adjusted (no CRP/comorbids) | RAR (per 1 SD) | 1.02 (0.93–1.11) | 0.734131201827635 |
| Main adjusted | RAR (per 1 SD) | 1.04 (0.95–1.14) | 0.4159827055814498 |
| Exclude CRP>10 mg/dL | RAR (per 1 SD) | 1.04 (0.95–1.15) | 0.39639212778725286 |
| Exclude ALT/AST/GGT ≥95th pct | RAR (per 1 SD) | 1.03 (0.93–1.14) | 0.5947043877115974 |
| Exclude RAR outliers (1–99%) | RAR (per 1 SD) | 1.09 (0.97–1.22) | 0.13466249429409666 |
| Log-transformed exposure | ln(RAR) | 1.75 (0.81–3.77) | 0.1544162441716742 |
| Quartiles (ref=Q1) |  | 1.36 (1.06–1.74) | 0.0160509388103976 |

Supplementary Table 4. Multivariable logistic regression analysis after exclusion of participants with clinical anemia.

| Exposure(RAR) | Non-adjusted model | Model I | Model II |
| --- | --- | --- | --- |
| Q1 (2.075-2.64) | Ref | Ref | Ref |
| Q2（2.64-2.81） | 1.49 (1.2~1.87) <0.001 | 1.31 (1.02~1.69) 0.036 | 1.18 (0.91~1.54) 0.217 |
| Q3（2.81-3.02） | 2 (1.6~2.49) <0.001 | 1.5 (1.15~1.94) 0.002 | 1.39 (1.06~1.83) 0.018 |
| Q4（3.02-7.43） | 1.9 (1.53~2.37) <0.001 | 1.49 (1.14~1.95) 0.003 | 1.33 (1~1.77) 0.053 |
| p for trend | <0.001 | 0.003 | 0.036 |

Model 1 adjust for: sex, age, race

Model 2 adjust for: sex, age, race, education level, marital status, PIR, alcohol, BMI, hypertension, diabetes, coronary heart disease, stroke, smoking.
